# Benchmarking metabolic surrogate indices against body mass index for left atrial diameter in atrial fibrillation: a two-cohort study

**DOI:** 10.3389/fendo.2026.1968894

**Published:** 2026-11-11

**Authors:** Tianyu Li, Jie Zheng, Jingxuan Lu, Jingru Yang, Dan Li, Zhiming Yu

**Affiliations:** 1 Department of Cardiology, the Affiliated Wuxi People’s Hospital of Nanjing Medical University, Wuxi People’s Hospital, Wuxi Medical Center, Nanjing Medical University, Wuxi, China; 2 Department of Endocrinology, Nanjing First Hospital, Nanjing Medical University, Nanjing, China

**Keywords:** atrial fibrillation, body mass index, commonality analysis, insulin resistance, left atrial diameter, metabolic surrogate indices, replication

## Abstract

**Background:**

Metabolic surrogate indices are increasingly used in atrial fibrillation (AF), but several algebraically incorporate body mass index (BMI). We examined whether their associations with left atrial diameter (LAD) provide information beyond a simple anthropometric benchmark.

**Methods:**

We analyzed 788 patients in a primary AF cohort and 813 in a replication cohort. Seven prespecified metabolic surrogate indices were compared with BMI using harmonized linear models. We evaluated adjusted associations and incremental R² (ΔR²), partitioned shared and unique ΔR² by commonality analysis, and quantified BMI-index ΔR² differences by paired bootstrap resampling. Sensitivity analyses addressed body-size indexing, additional height/BSA covariate adjustment, AF-phenotype stratification, and covariate specification.

**Results:**

Indices without BMI in their formulas—TyG, TG/HDL-C, TG/G, and CHG—added little ΔR² in either cohort (all <0.4 percentage points). SPISE, TyG-BMI, and METS-IR contributed 5.66, 4.79, and 4.45 percentage points in the primary cohort and 3.57, 3.51, and 3.35 percentage points in the replication cohort, versus 6.23 and 4.29 percentage points for BMI. Most ΔR² of the BMI-containing indices was shared with BMI, whereas index-unique components were small. Bootstrap comparisons supported numerically similar or greater ΔR² for BMI, although confidence intervals did not uniformly exclude zero. Height-adjusted raw-LAD models preserved the comparative pattern, whereas additional BSA adjustment substantially attenuated BMI-related associations, supporting a body-size/allometric component.

**Conclusions:**

Indices without BMI in their formulas added little ΔR² for contemporaneous LAD, whereas BMI-containing composites derived most of their ΔR² from variance shared with BMI. BMI provided numerically similar or greater ΔR², although paired bootstrap comparisons were not statistically distinguishable for all comparisons. Additional body-size covariate analyses indicated that a substantial component of these associations may reflect body-size/allometric structure. BMI should therefore be an explicit benchmark when evaluating metabolic surrogate indices for left atrial size.

## 1 Introduction

Atrial fibrillation (AF) is closely linked to cardiometabolic risk, and contemporary European and North American guidelines emphasize management of obesity and related metabolic conditions alongside conventional AF treatment (1, 2). Direct measurement of insulin sensitivity is rarely feasible in routine AF cohorts, so observational studies commonly use surrogate indices derived from fasting glucose, triglycerides, cholesterol fractions, and anthropometric measures (3).

The triglyceride-glucose (TyG) index is among the most widely studied metabolic surrogates in AF, although reported associations vary across populations and outcomes (4, 5). The central question for comparative studies, however, is not simply which derived index has the largest association, but whether it adds information beyond simpler measures already embedded in its construction.

A methodological problem arises because TyG-BMI, METS-IR, and SPISE algebraically incorporate BMI, whereas TyG, TG/HDL-C, TG/G, and CHG do not (6–12). Stronger performance of BMI-containing indices for body-size-related outcomes may therefore partly reflect formula construction. Direct benchmarking against BMI and partitioning shared versus unique ΔR² are needed to determine whether a more complex surrogate contributes information beyond a simple anthropometric benchmark.

This issue is particularly relevant for left atrial size because chamber dimensions depend on body habitus, and contemporary echocardiographic guidance favors volumetric and body-size-indexed assessment (13). We therefore conducted a two-cohort methodological benchmarking analysis using contemporaneous LAD as the primary continuous outcome. We examined whether indices without BMI in their formulas added meaningful ΔR², quantified shared and unique information for BMI-containing composites relative to BMI, and tested whether the comparative pattern persisted across body-size and alternative covariate specifications.

## 2 Materials and methods

### 2.1 Study design and populations

This observational analysis used a primary clinical AF cohort and a second, publicly available AF cohort for replication. The primary cohort was assembled at Wuxi People’s Hospital from January 2019 to December 2025. The source dataset contained 804 participants with AF. Three records with invalid adult age values were excluded, followed by 10 participants with invalid anthropometric/derived-BMI data and three participants with incomplete inputs required for the seven-index analysis, leaving 788 common complete cases (**Figure 1**). Quality-control flags for height and BMI were not mutually exclusive. This observational study was reported in accordance with the Strengthening the Reporting of Observational Studies in Epidemiology (STROBE) statement (14).

**Figure 1:**
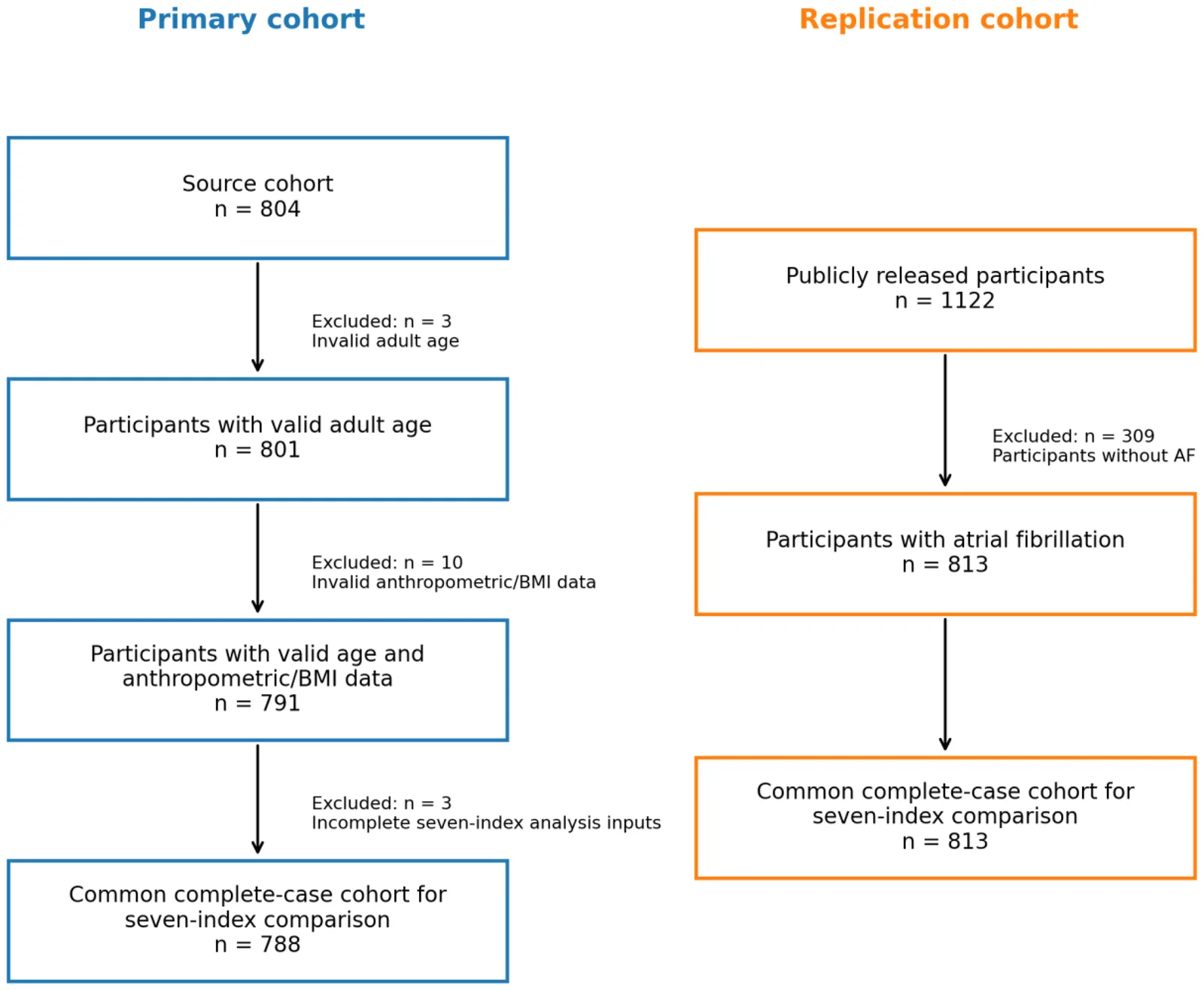
Study population and analytic sample flow.

The replication cohort was reconstructed from the patient-level supporting dataset of Li et al. (15), which included 1,122 hospitalized participants at the First Hospital of Jilin University between January 1, 2023 and February 29, 2024. We excluded 309 participants without AF and retained all 813 participants with paroxysmal or persistent AF because the variables required for the seven-index comparison were complete in that subgroup. Recalculated BMI, TyG, and TyG-BMI values were virtually identical to the released derived variables (**Supplementary Table S2D**). Patient-level age and AF duration were not present in the released supporting file and were not imputed from aggregate summaries.

For cross-cohort comparisons, we prespecified a harmonized core adjustment set available in both cohorts: sex, AF phenotype, hypertension, diabetes mellitus, coronary artery disease, prior stroke, and left ventricular ejection fraction (LVEF). Age and AF duration were available only in the primary cohort and were incorporated in dedicated bounding analyses. Smoking and drinking status were available in the replication cohort and were added in a separate sensitivity analysis.

### 2.2 Outcomes and clinical variables

The primary outcome was left atrial diameter (LAD), analyzed continuously in millimeters. The secondary outcome was a sex-specific diameter-defined left atrial enlargement (LAE) phenotype, defined as LAD >40 mm in men or >38 mm in women (13). Because LAE is a dichotomized representation of the same LAD measurement, it was treated as a secondary coarsened analysis rather than as an independent validation endpoint. Left atrial volume index was not available in both cohorts; contemporary chamber-quantification guidance favors volumetric assessment for routine evaluation of left atrial size (13).

Height and weight were used to calculate body surface area (BSA) using the Mosteller formula, BSA = sqrt[height(cm) × weight(kg)/3600] (16). To examine body-size dependence of the primary findings, we repeated the main models with LAD/BSA (mm/m²) and LAD/height (mm/m) as outcomes.

AF phenotype was categorized as paroxysmal or persistent. Hypertension, diabetes mellitus, coronary artery disease, and prior stroke were represented as binary clinical variables. In the primary cohort, fasting venous blood samples were used for measurement of fasting plasma glucose and triglycerides. In the replication cohort, the source report likewise documented fasting peripheral venous blood collection the following morning for triglycerides and fasting glucose (15). At Wuxi People’s Hospital, the institutional standard protocol measures LAD by two-dimensional echocardiography in the parasternal long-axis view at end-systole, following institutional practice based on American Society of Echocardiography chamber-quantification guidance (13). Rhythm at the time of echocardiographic acquisition was not recorded in the available analytic datasets and therefore could not be used as an adjustment or stratification variable. Reader-level reproducibility data and sufficiently detailed acquisition metadata for the replication cohort were unavailable, precluding full imaging-protocol harmonization across cohorts.

### 2.3 Metabolic surrogate indices

We evaluated seven prespecified metabolic surrogate indices. Glucose, triglycerides, total cholesterol, and HDL-C were converted from mmol/L to mg/dL when required by the published formulas. TyG was calculated as ln[(TG × FPG)/2] (6). TyG-BMI was calculated as TyG × BMI (7). METS-IR was calculated as ln(2 × FPG + TG) × BMI/ln(HDL-C) (8). CHG was calculated as ln[(TC × FPG)/(2 × HDL-C)] (9). SPISE was calculated in its original direction as 600 × HDL-C^0.185/(TG^0.2 × BMI^1.338), such that higher values indicate greater insulin sensitivity (10). TG/HDL-C and TG/G were calculated directly from their component concentrations (11, 12).

For interpretation, TyG, TG/HDL-C, TG/G, and CHG were classified as indices that do not algebraically incorporate BMI, whereas SPISE, TyG-BMI, and METS-IR algebraically incorporate BMI. This classification refers only to formula structure and does not imply biological independence from adiposity. All seven indices and BMI were standardized to cohort-specific Z scores for the primary comparative models. Per-IQR scaling was examined as an alternative exposure scale. Direct clamp-based insulin sensitivity was not available; the indices were therefore interpreted as pragmatic metabolic surrogates rather than interchangeable measurements of insulin resistance (3).

### 2.4 Statistical analysis

For the primary LAD analysis, each standardized index and BMI were entered separately into linear regression models containing the harmonized core covariates. Effect estimates are reported as adjusted LAD differences per 1-SD increase. For each marker, we calculated incremental R² (ΔR²) beyond the clinical covariate model. Benjamini-Hochberg false-discovery-rate (FDR) correction was applied across the seven metabolic surrogate indices within each testing family (17). BMI was a prespecified benchmark and was intentionally excluded from that multiplicity family; this analytic asymmetry was prespecified, and BMI nominal significance was not used to claim superiority.

Because high correlation between BMI and BMI-containing indices can destabilize coefficients in joint models, we did not use BMI-adjusted index coefficients as the primary evidence for separable information. Instead, for SPISE, TyG-BMI, and METS-IR we used commonality analysis to partition ΔR² beyond the clinical model into variance shared by BMI and the index, BMI-unique variance, and index-unique variance (18). We quantified direct head-to-head uncertainty using paired participant-level bootstrap resampling (3,000 resamples) of the difference ΔR²_BMI − ΔR²_index. Variance inflation factors (VIFs) and tolerance were reported for joint BMI-index models, and the corresponding coefficients were retained descriptively only.

Sensitivity analyses addressed several design concerns. First, all eight markers were analyzed against LAD/BSA and LAD/height. Second, to avoid reliance on ratio indexing, unindexed LAD models were refit with height or BSA entered as an additional covariate to the harmonized core model (**Supplementary Table S15**). Third, the primary cohort was refit using the harmonized core set, core + age, and core + age + AF duration to bound the effect of covariates unavailable in the replication cohort. Fourth, reduced models that omitted AF phenotype and LVEF were examined to assess sensitivity to potential downstream-variable adjustment. Fifth, the LAD models were repeated separately within paroxysmal and persistent AF strata, omitting AF phenotype from the stratum-specific covariate set; this analysis was treated only as a phenotype-based partial proxy and not as evidence of actual rhythm at echocardiographic acquisition (**Supplementary Table S16**). Additional analyses excluded participants with diabetes, used HC3 heteroscedasticity-consistent standard errors (19), added smoking and drinking to the replication model, and rescaled exposures per IQR.

Restricted cubic splines with four knots were used for BMI, SPISE, TyG-BMI, and METS-IR; overall and nonlinearity tests were obtained from nested-model comparisons, and curves were displayed over common central support to limit tail extrapolation (20). Exploratory AF-phenotype interactions were tested with marker × phenotype terms and FDR correction. Primary-cohort conditional quantile regression was performed at the 0.25, 0.50, 0.75, and 0.90 LAD quantiles (21). Spearman correlation matrices were reported as descriptive supplementary analyses rather than as evidence of independent information.

The secondary diameter-defined LAE analysis used logistic regression. We report odds ratios per 1-SD increase, AUC, Brier score, and changes in AUC relative to the clinical-only model. Paired AUC comparisons used the DeLong method (22). Apparent calibration was examined graphically, and 1,000 bootstrap resamples were used for optimism-corrected AUC, Brier score, calibration slope, and calibration intercept (23). Decision-curve analysis was not performed because LAE was a contemporaneous dichotomization of LAD rather than a prospective decision outcome. A separate 1,000-resample bootstrap assessed rank stability among the seven surrogate indices for continuous-LAD association metrics (absolute standardized β and ΔR²) and, secondarily, for ΔAUC in the diameter-defined LAE analysis. All analyses were conducted in R version 4.5.2. Two-sided P < 0.05 was considered statistically significant.

### 2.5 Ethics

The primary cohort was approved by the Ethics Committee of Wuxi People’s Hospital (approval number KY26162), which waived the requirement for written informed consent because of the retrospective nature of the study. The replication analysis used de-identified data publicly released with the original PLOS One report (15).

## 3 Results

### 3.1 Study population and cohort characteristics

The primary source cohort contained 804 participants. After sequential exclusions for invalid age, invalid anthropometric/derived-BMI data, and incomplete seven-index inputs, 788 participants remained. The replication dataset contained 1,122 participants; excluding 309 without AF left 813 participants with paroxysmal or persistent AF (**Figure 1**).

In the primary cohort, median age was 63.0 years [57.0, 70.0], 57.7% were men, and 41.1% had persistent AF. Mean BMI was 25.17 ± 3.31 kg/m², mean LAD was 40.74 ± 5.54 mm, and 58.6% met the diameter-defined LAE criterion. In the replication cohort, 61.1% were men and 49.7% had persistent AF; mean BMI was 26.06 ± 3.45 kg/m², mean LAD was 39.95 ± 6.09 mm, and 47.7% had LAE. Mean BSA was 1.80 ± 0.18 m² and 1.84 ± 0.18 m² in the primary and replication cohorts, respectively (**Table 1**).

**Table 1:** Cohort characteristics.

Characteristic	Primary cohort (n=788)	Replication cohort (n=813)
Age, years, median [IQR]	63.0 [57.0, 70.0]	—†
Male sex	455 (57.7%)	497 (61.1%)
Height, cm	166.94 ± 8.13	167.61 ± 7.94
Weight, kg	70.31 ± 11.63	73.42 ± 12.25
BSA, m²	1.80 ± 0.18	1.84 ± 0.18
BMI, kg/m²	25.17 ± 3.31	26.06 ± 3.45
Persistent AF	324 (41.1%)	404 (49.7%)
AF duration, months, median [IQR]	24.0 [8.0, 60.0]	—†
Hypertension	476 (60.4%)	429 (52.8%)
Diabetes mellitus	124 (15.7%)	178 (21.9%)
Coronary artery disease	90 (11.4%)	178 (21.9%)
Prior stroke	70 (8.9%)	91 (11.2%)
LVEF, %	62.4 ± 3.9	59.3 ± 7.9
LAD, mm	40.74 ± 5.54	39.95 ± 6.09
LAE	462 (58.6%)	388 (47.7%)
Fasting plasma glucose, mmol/L	5.14 ± 0.99	6.00 ± 1.83
Triglycerides, mmol/L, median [IQR]	1.32 [0.99, 1.76]	1.54 [1.19, 2.10]
HDL-C, mmol/L	1.09 ± 0.24	1.06 ± 0.24
Total cholesterol, mmol/L	4.12 ± 0.98	4.43 ± 1.04

Values are mean ± SD, median [IQR], or n (%), as appropriate. BSA was calculated using the Mosteller formula. †Patient-level age and AF duration were unavailable in the replication public AF dataset; aggregate summaries were not used to impute individual-level values.

### 3.2 Comparative associations of BMI and metabolic surrogate indices with LAD

The most informative contrast was between indices that did and did not algebraically incorporate BMI (**Figure 2**; **Table 2**). Among the four indices without BMI in their formulas, ΔR² was small in both cohorts. In the primary cohort, ΔR² values were 0.36 percentage points for TyG, 0.20 for TG/HDL-C, 0.06 for TG/G, and 0.04 for CHG. The corresponding replication-cohort values were 0.12, <0.01, 0.03, and 0.18 percentage points. None of these four indices showed a robust cross-cohort association after FDR correction.

**Figure 2:**
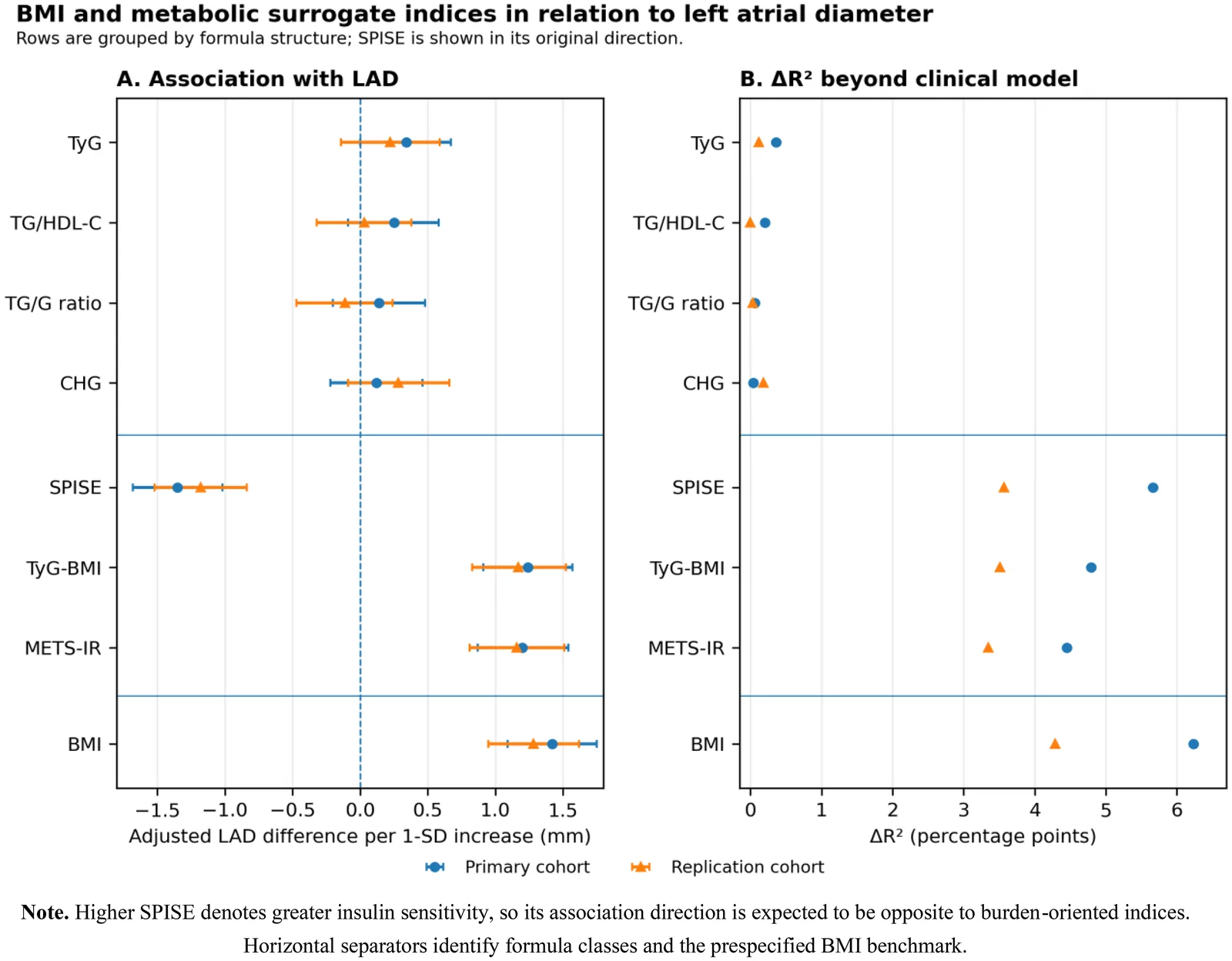
**(A)** Adjusted associations of BMI and metabolic surrogate indices with left atrial diameter (LAD), expressed as adjusted LAD differences per 1-SD increase with 95% confidence intervals. **(B)** Incremental explained variance (ΔR^2^, percentage points) beyond the harmonized clinical model for each marker in the primary and replication cohorts.

**Table 2:** Comparative associations of BMI and metabolic surrogate indices with left atrial diameter.

Cohort	Marker	Adjusted LAD difference per 1-SD (95% CI), mm	Nominal P	FDR P	ΔR², percentage points
Primary cohort	TyG	0.34 (0.00, 0.67)	0.049	0.086	0.36
Primary cohort	TG/HDL-C	0.25 (-0.09, 0.58)	0.146	0.204	0.20
Primary cohort	TG/G ratio	0.14 (-0.20, 0.48)	0.413	0.482	0.06
Primary cohort	CHG	0.12 (-0.22, 0.46)	0.494	0.494	0.04
Primary cohort	SPISE	-1.35 (-1.68, -1.02)	<0.001	<0.001	5.66
Primary cohort	TyG-BMI	1.24 (0.91, 1.57)	<0.001	<0.001	4.79
Primary cohort	METS-IR	1.20 (0.87, 1.54)	<0.001	<0.001	4.45
Primary cohort	BMI	1.42 (1.09, 1.75)	<0.001	—‡	6.23
Replication cohort	TyG	0.22 (-0.14, 0.59)	0.230	0.321	0.12
Replication cohort	TG/HDL-C	0.03 (-0.32, 0.38)	0.885	0.885	<0.01
Replication cohort	TG/G ratio	-0.11 (-0.47, 0.24)	0.532	0.621	0.03
Replication cohort	CHG	0.28 (-0.09, 0.66)	0.140	0.246	0.18
Replication cohort	SPISE	-1.18 (-1.52, -0.84)	<0.001	<0.001	3.57
Replication cohort	TyG-BMI	1.17 (0.83, 1.52)	<0.001	<0.001	3.51
Replication cohort	METS-IR	1.16 (0.81, 1.51)	<0.001	<0.001	3.35
Replication cohort	BMI	1.28 (0.95, 1.62)	<0.001	—‡	4.29

Cross-cohort models use the harmonized core covariate set: sex, AF phenotype, hypertension, diabetes mellitus, coronary artery disease, prior stroke, and LVEF. All markers are standardized within cohort. BH-FDR correction is applied to the seven metabolic surrogate indices only. ‡ BMI is a prespecified benchmark and is intentionally outside that multiplicity family; its significance is not used to claim superiority.

By contrast, the three BMI-containing indices showed substantially larger associations and ΔR² values. In the primary cohort, SPISE was inversely associated with LAD (β = -1.35 mm per SD, 95% CI -1.68 to -1.02; ΔR² = 5.66 percentage points), whereas TyG-BMI and METS-IR were positively associated (β = 1.24 and 1.20 mm; ΔR² = 4.79 and 4.45 percentage points). In the replication cohort, the corresponding ΔR² values were 3.57, 3.51, and 3.35 percentage points. BMI itself produced ΔR² values of 6.23 percentage points in the primary cohort and 4.29 percentage points in the replication cohort.

### 3.3 Shared and unique explained variance relative to BMI

The BMI-containing indices were highly collinear with BMI in joint models. VIFs ranged from 3.21 to 7.85 in the primary cohort and from 3.92 to 7.39 in the replication cohort, with the highest values for TyG-BMI (**Supplementary Table S7A**). Joint coefficients were correspondingly unstable: for example, TyG-BMI and METS-IR changed direction in the primary BMI-index models, with wide confidence intervals spanning zero (**Supplementary Table S7B**). These coefficients were therefore not interpreted as evidence of separable biological effects.

Commonality decomposition showed that most of each BMI-containing index’s ΔR² was shared with BMI (**Figure 3**; **Table 3**). In the primary cohort, shared components were 5.33 percentage points for BMI + SPISE, 4.66 for BMI + TyG-BMI, and 4.38 for BMI + METS-IR; index-unique components were 0.34, 0.13, and 0.07 percentage points. In the replication cohort, shared components were 3.52, 3.50, and 3.35 percentage points, whereas index-unique components were 0.05, 0.01, and <0.01 percentage points.

**Figure 3:**
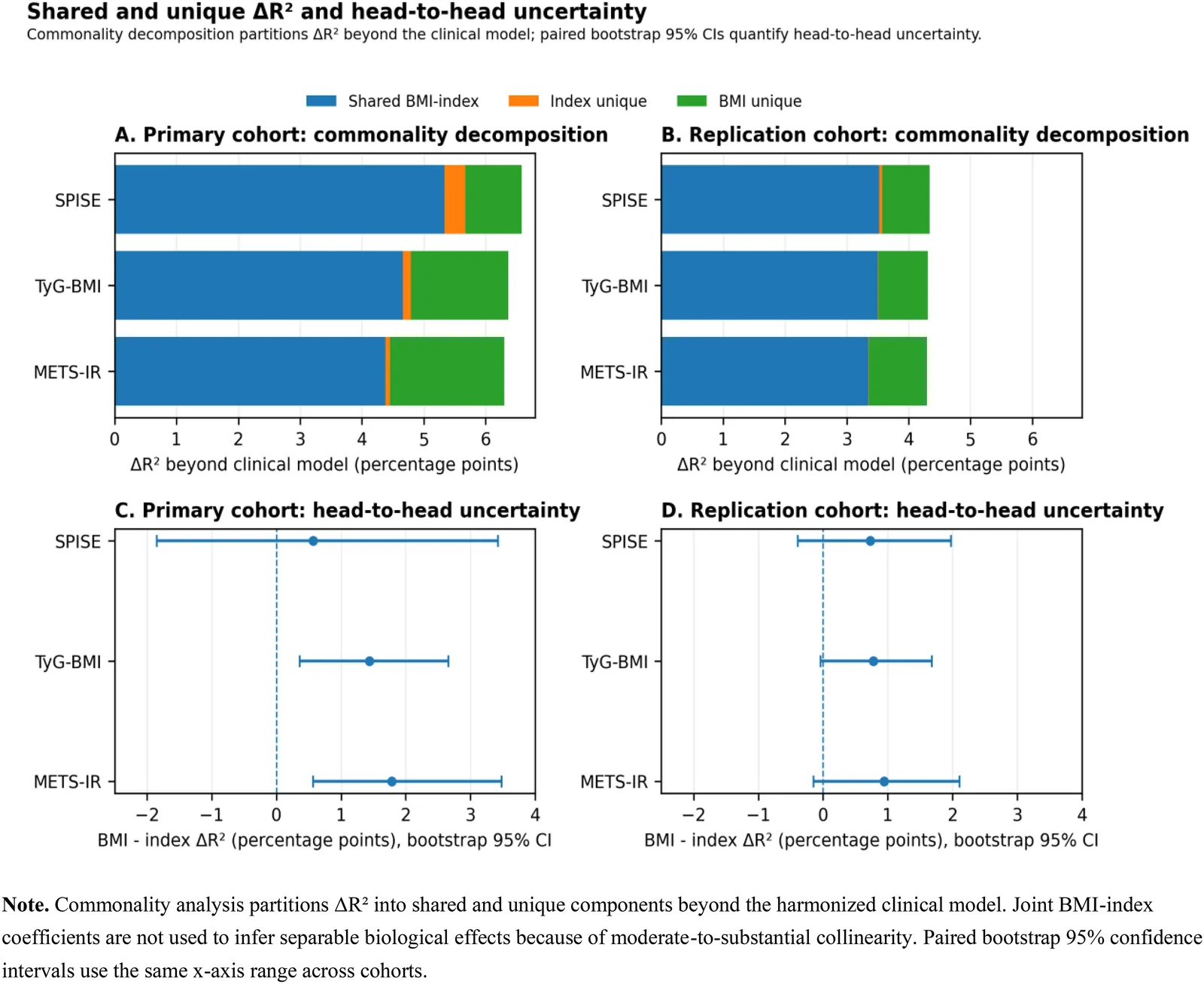
Shared and unique ΔR² and head-to-head uncertainty. **(A)** Commonality decomposition in the primary cohort. **(B)** Commonality decomposition in the replication cohort. **(C)** Paired bootstrap comparisons of BMI minus index ΔR² in the primary cohort. **(D)** Corresponding paired bootstrap comparisons in the replication cohort. Commonality analysis partitions incremental explained variance (ΔR²) beyond the harmonized clinical model into components shared by BMI and each index, unique to BMI, and unique to the index. Joint BMI-index coefficients are not used to infer separable biological effects because of moderate-to-substantial collinearity. Paired bootstrap 95% confidence intervals use the same x-axis range across cohorts.

**Table 3:** Head-to-head BMI benchmarking: ΔR², bootstrap uncertainty, and commonality decomposition.

Cohort	BMI-containing index	BMI ΔR², pp	Index ΔR², pp	BMI - index ΔR² (95% bootstrap CI), pp	Shared BMI-index ΔR², pp	BMI-unique ΔR², pp	Index-unique ΔR², pp
Primary cohort	SPISE	6.23	5.66	0.57 (-1.85, 3.43)	5.33	0.91	0.34
Primary cohort	TyG-BMI	6.23	4.79	1.44 (0.36, 2.66)	4.66	1.58	0.13
Primary cohort	METS-IR	6.23	4.45	1.78 (0.57, 3.48)	4.38	1.85	0.07
Replication cohort	SPISE	4.29	3.57	0.73 (-0.39, 1.98)	3.52	0.77	0.05
Replication cohort	TyG-BMI	4.29	3.51	0.78 (-0.04, 1.68)	3.50	0.80	0.01
Replication cohort	METS-IR	4.29	3.35	0.94 (-0.15, 2.11)	3.35	0.94	<0.01

Shared/unique components are commonality partitions of ΔR² beyond the harmonized clinical model. Positive BMI-index ΔR² differences favor BMI. Percentile bootstrap confidence intervals use paired participant-level resampling (B = 3,000). Components may not sum exactly to displayed standalone ΔR² values because of rounding; nonzero components below 0.01 percentage points are reported as <0.01. VIF/tolerance and descriptive joint BMI-index coefficients are reported separately in **Supplementary Tables S7A and S7B**.

Paired bootstrap comparisons quantified uncertainty in direct ΔR² differences. In the primary cohort, BMI minus index ΔR² differences were 0.57 percentage points for SPISE (95% CI -1.85 to 3.43), 1.44 for TyG-BMI (0.36 to 2.66), and 1.78 for METS-IR (0.57 to 3.48). In the replication cohort, the differences were 0.73 (-0.39 to 1.98), 0.78 (-0.04 to 1.68), and 0.94 (-0.15 to 2.11) percentage points, respectively. Thus, BMI yielded numerically similar or greater ΔR², but the head-to-head difference was not statistically distinguishable for every comparison.

### 3.4 Body-size-indexed sensitivity analyses

Body-size indexing materially changed interpretation when BSA was used as the denominator (**Figure 4**; **Supplementary Table S4**). For LAD/BSA, BMI ΔR² was 2.67 percentage points in the primary cohort and 4.77 in the replication cohort. BMI, TyG-BMI, and METS-IR coefficients reversed direction, whereas SPISE became positive.

**Figure 4:**
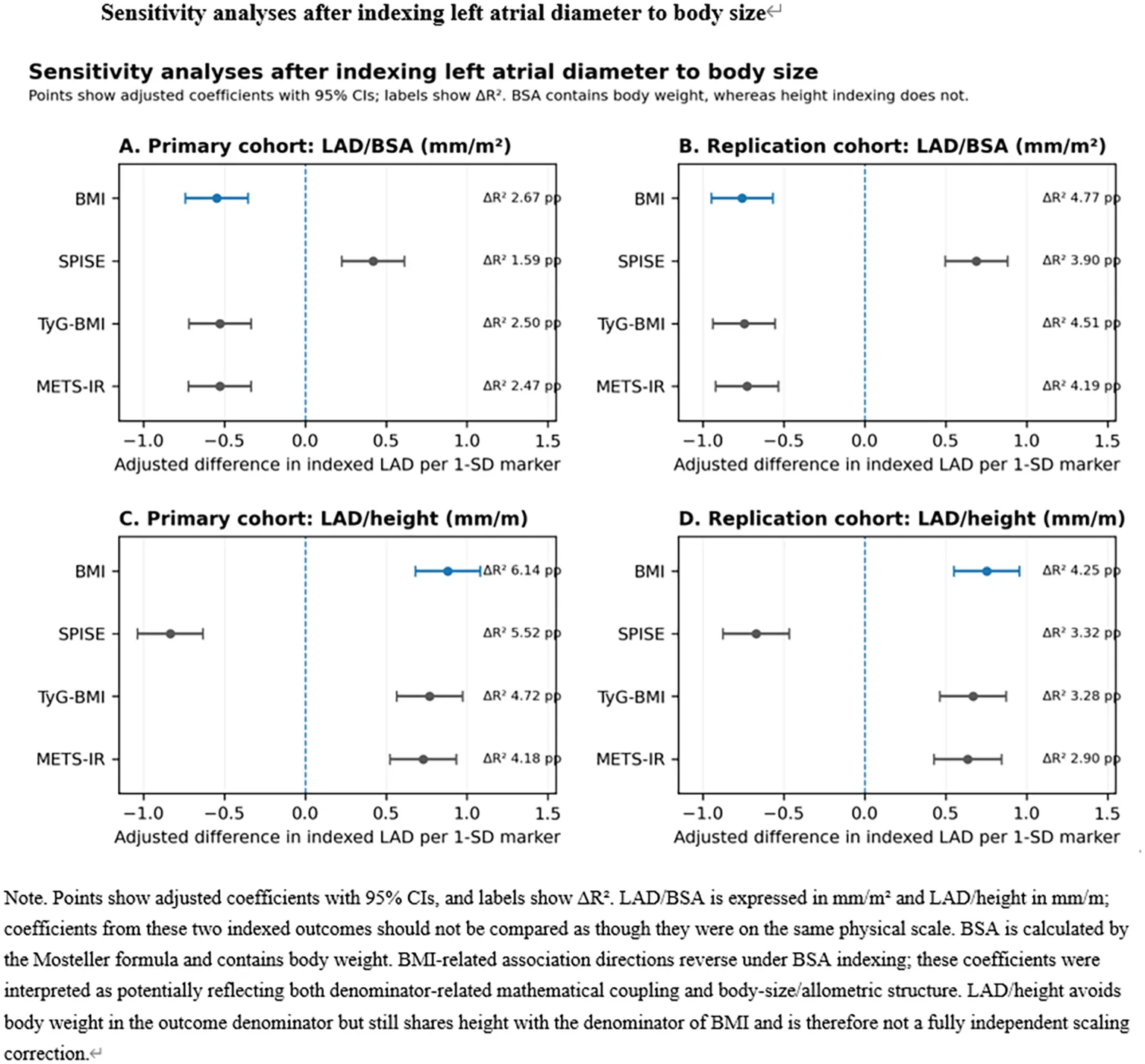
Sensitivity analyses after indexing left atrial diameter to body size. **(A)** LAD/BSA analysis in the primary cohort. **(B)** LAD/BSA analysis in the replication cohort. **(C)** LAD/height analysis in the primary cohort. **(D)** LAD/height analysis in the replication cohort. Points show adjusted coefficients with 95% CIs, and labels show ΔR². LAD/BSA is expressed in mm/m² and LAD/height in mm/m; coefficients from these two indexed outcomes should not be compared as though they were on the same physical scale. BSA is calculated using the Mosteller formula and incorporates body weight. BMI-related association directions reverse under BSA indexing; these coefficients may reflect both denominator-related mathematical coupling and body-size/allometric structure. LAD/height avoids body weight in the outcome denominator but still shares height with the denominator of BMI and is therefore not a fully independent scaling correction.

Height indexing preserved the original association directions and comparative ordering. For LAD/height, BMI ΔR² was 6.14 percentage points in the primary cohort and 4.25 in the replication cohort; SPISE contributed 5.52 and 3.32, TyG-BMI 4.72 and 3.28, and METS-IR 4.18 and 2.90 percentage points. The four indices without BMI in their formulas again contributed little explained variance. However, LAD/height is not mathematically independent of BMI because height also appears in the denominator of BMI.

To avoid ratio-indexing artifacts, we retained unindexed LAD as the outcome and entered height or BSA as an additional covariate (**Supplementary Table S15**). With height adjustment, ΔR² values for BMI, SPISE, TyG-BMI, and METS-IR were 6.32, 5.73, 4.85, and 4.47 percentage points in the primary cohort and 4.22, 3.42, 3.38, and 3.12 in the replication cohort. With BSA adjustment, the corresponding values attenuated to 1.09, 1.18, 0.48, and 0.38 percentage points in the primary cohort and 0.23, 0.18, 0.08, and 0.07 in the replication cohort. This attenuation is consistent with a substantial body-size component.

### 3.5 Covariate and exposure-scaling sensitivity analyses

In the primary cohort, adding age and then AF duration did not materially alter the comparative hierarchy (**Supplementary Table S5**). BMI ΔR² changed from 6.23 percentage points in the harmonized core model to 6.52 after adding age and 6.46 after adding both age and AF duration. Corresponding values for SPISE were 5.66, 6.29, and 6.26; for TyG-BMI, 4.79, 5.12, and 5.08; and for METS-IR, 4.45, 4.85, and 4.83 percentage points.

Reduced and minimally adjusted models preserved the broader separation between BMI/BMI-containing composites and the weaker non-BMI formula indices, although some individual estimates among the latter were model-sensitive, particularly in the replication cohort (**Supplementary Table S6**). In the reduced models omitting AF phenotype and LVEF, ΔR² values for BMI, SPISE, TyG-BMI, and METS-IR were 10.39, 8.96, 7.94, and 7.43 percentage points in the primary cohort and 4.38, 1.42, 1.95, and 2.03 in the replication cohort; the four indices without BMI in their formulas remained at or below 0.53 and 0.83 percentage points, respectively. Excluding diabetes and using HC3 robust standard errors did not materially change the leading hierarchy, and adding smoking and drinking to the replication model produced only modest changes (**Supplementary Table S13**; **Supplementary Figure S7**). Per-IQR scaling yielded the same comparative pattern as the per-SD analysis (**Supplementary Table S14**).

### 3.6 Dose-response and exploratory heterogeneity

Restricted cubic splines supported overall associations for BMI, SPISE, TyG-BMI, and METS-IR in both cohorts (all P overall < 0.001; **Supplementary Figure S2**; **Supplementary Table S8**). Evidence of nonlinearity was present for BMI (P = 0.002 primary; P = 0.031 replication), TyG-BMI (P = 0.002; P = 0.003), and METS-IR (P = 0.002; P < 0.001), whereas SPISE was approximately linear (P for nonlinearity = 0.897 and 0.127).

No AF-phenotype interaction met the prespecified FDR threshold in either cohort (**Supplementary Figure S3**; **Supplementary Table S9**). In the primary age- and AF-duration-adjusted analysis, TyG-BMI and METS-IR interaction FDR P values were both 0.052, but the pattern was not reproduced in the replication cohort. In phenotype-stratified models (**Supplementary Table S16**), ΔR² values for BMI, SPISE, TyG-BMI, and METS-IR were 5.06, 5.12, 3.54, and 3.30 percentage points in primary paroxysmal AF and 13.51, 10.87, 11.40, and 10.55 in primary persistent AF. Corresponding replication-cohort values were 6.65, 6.43, 5.82, and 5.83 in paroxysmal AF and 3.93, 3.31, 3.03, and 2.86 in persistent AF. The four non-BMI formula indices remained weaker within each stratum. AF phenotype was interpreted only as a partial proxy and not as documentation of rhythm during echocardiographic acquisition. Quantile regression showed that the associations of SPISE, TyG-BMI, and METS-IR were present across the LAD distribution rather than being confined to the largest atria, whereas estimates for the non-BMI formula indices were smaller and less consistent (**Supplementary Figure S4**; **Supplementary Table S10**). Descriptive correlation matrices are provided in **Supplementary Figure S1** and **Supplementary Tables S3A-S3D**.

### 3.7 Secondary diameter-defined LAE analyses

Diameter-defined LAE was analyzed as a secondary dichotomization of LAD rather than as an independent outcome. In the primary cohort, AUCs were 0.797 for Clinical + BMI, 0.794 for Clinical + SPISE, 0.795 for Clinical + TyG-BMI, and 0.791 for Clinical + METS-IR. In the replication cohort, the corresponding AUCs were 0.812, 0.815, 0.813, and 0.810 (**Supplementary Table S11A**; **Supplementary Figure S5**). Bootstrap optimism correction produced only modest changes in performance and calibration (**Supplementary Table S11B**). Rank-stability analysis again favored SPISE and TyG-BMI for association-based metrics, but rank probabilities varied by metric (**Supplementary Figure S6**; **Supplementary Table S12**). No decision-curve analysis was performed.

## 4 Discussion

### 4.1 Principal findings

This two-cohort study supports a methodological interpretation of metabolic surrogate ranking in AF. Indices without BMI in their formulas added little ΔR², whereas BMI-containing composites showed stronger associations but shared most of their explanatory information with BMI. BMI provided numerically similar or greater ΔR², although not every paired comparison was statistically distinguishable. These findings support direct benchmarking of complex surrogates against simpler constituent or anthropometric measures.

### 4.2 Formula composition and the limits of surrogate ranking

Formula construction is central to interpretation. TyG-BMI multiplies TyG by BMI, METS-IR includes BMI directly, and SPISE contains BMI^1.338 in the denominator (7, 8, 10). Their stronger associations with a body-size-related phenotype are therefore partly expected. By contrast, the four non-BMI indices provide a cleaner test of lipid-glucose information beyond anthropometry. The consistently small ΔR² values of the non-BMI indices indicate weak alignment with contemporaneous LAD after clinical adjustment in these AF cohorts. This outcome-specific finding does not imply that TyG or related markers lack cardiometabolic relevance; it indicates that, for LAD, formula complexity and embedded anthropometry were more closely related to explanatory strength.

### 4.3 Shared versus unique information relative to BMI

Joint BMI-index coefficients were difficult to interpret because of substantial collinearity. VIFs were highest for the BMI/TyG-BMI pair, and some joint coefficients changed sign. We therefore relied on commonality decomposition to describe overlap rather than on conditional coefficients as evidence of separable effects. Across both cohorts, most ΔR² of the BMI-containing indices was shared with BMI, while index-unique components were small and approached zero in the replication cohort. This pattern argues against interpreting the composites as clearly additive to BMI. Paired bootstrap comparisons added uncertainty information. Some primary-cohort BMI-index differences excluded zero, whereas the BMI-SPISE comparison and all replication-cohort comparisons did not. Thus, BMI provided numerically similar or greater ΔR², but universal statistical superiority is not supported.

### 4.4 Body-size indexing and interpretation of LAD

Body-size scaling remains an important interpretive issue because chamber dimensions depend on body habitus (13). The coefficient reversals under LAD/BSA may reflect denominator-related mathematical coupling, but they are also compatible with a substantial body-size/allometric component of the BMI-LAD association. Height indexing preserved the original direction and ordering, but it does not fully resolve this issue because height also appears in the denominator of BMI. The raw-LAD sensitivity models therefore provide a complementary assessment. Additional height adjustment left the comparative pattern largely unchanged, whereas additional BSA adjustment substantially attenuated the incremental contribution of BMI and the BMI-containing composites, especially in the replication cohort (**Supplementary Table S15**). This attenuation supports an important body-size component, while its magnitude should still be interpreted cautiously because BSA itself incorporates body weight and is not mathematically independent of BMI. LAD is a one-dimensional measure of left atrial size, whereas contemporary echocardiographic guidance favors volumetric assessment (13). The findings should therefore be interpreted as associations with contemporaneous left atrial size rather than as evidence of longitudinal atrial remodeling.

### 4.5 Dose-response patterns and exploratory heterogeneity

Spline analyses indicated nonlinearity for BMI, TyG-BMI, and METS-IR but not SPISE. These patterns are descriptive and should not be interpreted mechanistically in this cross-sectional study. AF-phenotype interactions did not survive FDR correction, and the near-threshold primary interactions for TyG-BMI and METS-IR were not reproduced in the replication cohort. Phenotype-stratified models retained the broad contrast between BMI/BMI-containing indices and the non-BMI formula indices, but AF phenotype cannot establish the actual rhythm present during echocardiographic acquisition. Quantile regression suggested that the leading BMI-containing indices were associated with LAD across the distribution. These exploratory analyses did not alter the primary benchmarking conclusion.

### 4.6 Index proliferation and surrogate evaluation

These findings illustrate a broader problem of index proliferation in cardiometabolic research. Adding inputs or algebraic transformations can strengthen apparent associations without adding much unique information, particularly when candidate indices share glucose, triglycerides, HDL-C, and BMI.

Surrogate evaluation should therefore separate three questions. Is the marker associated with the outcome? Does it improve model fit beyond a clinically relevant simple benchmark? How much of its information is unique rather than shared with its constituents? Formula classification, direct benchmarking, uncertainty estimates, and variance partitioning provide complementary answers.

### 4.7 Strengths and limitations

Strengths include use of the same prespecified indices in two AF cohorts, harmonized core adjustment, formula-based classification, commonality decomposition, paired bootstrap uncertainty, and multiple sensitivity analyses.

The study is observational and primarily cross-sectional, so temporal and causal interpretations are not supported. Patient-level age and AF duration were unavailable in the replication cohort; primary-cohort bounding analyses were reassuring but cannot eliminate residual confounding in the second cohort. Some weaker non-BMI associations were adjustment-sensitive, so the second cohort should be viewed as replication of the comparative pattern rather than validation of identically adjusted effects.

LAD is an incomplete measure of atrial structure, and the secondary LAE phenotype is a dichotomization of the same measurement rather than an independent endpoint. Left atrial volume index would provide more complete structural phenotyping (13); future studies could additionally evaluate left atrial strain. The primary institution’s standard LAD protocol was confirmed, but rhythm at the time of echocardiographic acquisition and reader-level reproducibility data were unavailable, and sufficiently detailed acquisition metadata could not be harmonized for the replication cohort. AF-phenotype-stratified analyses therefore provide only a partial phenotype-based proxy and cannot substitute for documented rhythm at acquisition.

Finally, none of the seven indices is a direct measure of insulin sensitivity (3), and several share closely related lipid-glucose inputs (6–12). Fasting venous blood sampling for glucose and triglycerides was documented in both cohorts (15), but this does not convert the indices into direct physiological measurements. These limitations constrain mechanistic interpretation; within those constraints, the results still inform how such surrogates should be benchmarked.

### 4.8 Clinical and research implications

BMI does not directly characterize body composition or regional adiposity (24), so these findings do not imply that BMI replaces metabolic assessment. Rather, BMI should be used as an explicit benchmark when a surrogate algebraically contains or closely tracks adiposity.

Future studies should test whether metabolic surrogates improve longitudinal models or imaging phenotypes beyond simple anthropometric and laboratory measures, ideally in cohorts with standardized volumetric or strain imaging, complete age and rhythm data, and more direct metabolic phenotyping.

## 5 Conclusion

Across two AF cohorts, indices that did not algebraically incorporate BMI added little ΔR² for contemporaneous LAD. BMI-containing indices showed stronger associations, but most of their ΔR² was shared with BMI. BMI provided numerically similar or greater ΔR², although paired bootstrap comparisons were not statistically distinguishable for all comparisons. Additional body-size covariate analyses indicated that a substantial component of these associations may reflect body-size/allometric structure. These findings support explicit benchmarking against simple anthropometric measures and caution against interpreting stronger associations as unique metabolic information.

## Data Availability

The de-identified replication dataset is publicly available as the Supporting Information accompanying Li et al. (15). Data from the primary clinical cohort are available from the corresponding author upon reasonable request, subject to applicable institutional and ethical restrictions.
