# Supplementary material for: Benchmarking metabolic surrogate indices against body mass index for left atrial diameter in atrial fibrillation: a two-cohort study
Source: Front Endocrinol (Lausanne). 2026 Nov 11;17:1968894. doi: 10.3389/fendo.2026.1968894 (PMC13752438; doi:10.3389/fendo.2026.1968894)
Supplement: Supplementary file 1 [file SupplementaryFile1.docx]

# Supplementary Tables

**Table S1. Definitions and formula classification of metabolic surrogate indices**

| **Index** | **Formula** | **Input units** | **BMI algebraically included** | **Direction for analysis** | **Reference** |
| --- | --- | --- | --- | --- | --- |
| TyG | ln[(TG × FPG)/2] | TG and FPG in mg/dL | No | Higher = greater presumed metabolic/IR burden | PMID 19067533 |
| TG/HDL-C | TG / HDL-C | Both in mg/dL | No | Higher = greater presumed metabolic/IR burden | PMID 21632070 |
| TG/G ratio | TG / FPG | Both in mg/dL | No | Higher = greater presumed metabolic/IR burden | DOI 10.1016/j.dsx.2020.08.017 |
| CHG | ln[(TC × FPG)/(2 × HDL-C)] | TC, FPG and HDL-C in mg/dL | No | Higher = greater CHG value; not treated as clamp-validated IR | DOI 10.1111/jdi.14343 |
| SPISE | 600 × HDL-C^0.185 / (TG^0.2 × BMI^1.338) | HDL-C/TG in mg/dL; BMI kg/m² | Yes | Higher = greater insulin sensitivity | DOI 10.1373/clinchem.2016.257436 |
| TyG-BMI | TyG × BMI | TyG dimensionless; BMI kg/m² | Yes | Higher = greater presumed metabolic/IR burden | PMID 26930652 |
| METS-IR | ln(2×FPG + TG) × BMI / ln(HDL-C) | FPG/TG/HDL-C in mg/dL; BMI kg/m² | Yes | Higher = greater presumed metabolic/IR burden | PMID 29535168 |

**Note.** SPISE is reported in its original direction; higher SPISE denotes greater insulin sensitivity.

**Table S2A. Quality-control summary**

| **Cohort** | **QC item** | **n** |
| --- | --- | --- |
| Primary cohort | Implausible adult age | 3 |
| Primary cohort | Implausible height | 5 |
| Primary cohort | Invalid anthropometric/derived BMI | 10 |
| Primary cohort | Common complete-case cohort | 788 |
| Replication cohort | Publicly released participants | 1122 |
| Replication cohort | Participants with atrial fibrillation | 813 |
| Replication cohort | Common complete-case cohort | 813 |

**Table S2B. Missingness of key variables**

| **Cohort** | **Variable** | **Missing, n** | **Missing, %** | **Denominator** |
| --- | --- | --- | --- | --- |
| Primary cohort | BMI | 10 | 1.2 | 804 |
| Primary cohort | FPG | 3 | 0.4 | 804 |
| Primary cohort | TG | 3 | 0.4 | 804 |
| Primary cohort | HDL-C | 3 | 0.4 | 804 |
| Primary cohort | LAD | 0 | 0.0 | 804 |
| Primary cohort | LAE | 0 | 0.0 | 804 |
| Primary cohort | TyG | 3 | 0.4 | 804 |
| Primary cohort | TyG-BMI | 13 | 1.6 | 804 |
| Primary cohort | METS-IR | 13 | 1.6 | 804 |
| Primary cohort | CHG | 3 | 0.4 | 804 |
| Primary cohort | SPISE | 13 | 1.6 | 804 |
| Primary cohort | TG/HDL-C | 3 | 0.4 | 804 |
| Primary cohort | TG/G ratio | 3 | 0.4 | 804 |
| Replication cohort | BMI | 0 | 0.0 | 813 |
| Replication cohort | FPG | 0 | 0.0 | 813 |
| Replication cohort | TG | 0 | 0.0 | 813 |
| Replication cohort | HDL-C | 0 | 0.0 | 813 |
| Replication cohort | LAD | 0 | 0.0 | 813 |
| Replication cohort | LAE | 0 | 0.0 | 813 |
| Replication cohort | TyG | 0 | 0.0 | 813 |
| Replication cohort | TyG-BMI | 0 | 0.0 | 813 |
| Replication cohort | METS-IR | 0 | 0.0 | 813 |
| Replication cohort | CHG | 0 | 0.0 | 813 |
| Replication cohort | SPISE | 0 | 0.0 | 813 |
| Replication cohort | TG/HDL-C | 0 | 0.0 | 813 |
| Replication cohort | TG/G ratio | 0 | 0.0 | 813 |

**Table S2C. Analytic sample flow**

| **Cohort** | **Step** | **n** |
| --- | --- | --- |
| Primary cohort | Source cohort | 804 |
| Primary cohort | Participants with valid adult age | 801 |
| Primary cohort | Participants with valid age and anthropometric/BMI data | 791 |
| Primary cohort | Common complete-case cohort | 788 |
| Replication cohort | Publicly released participants | 1122 |
| Replication cohort | Participants with atrial fibrillation | 813 |
| Replication cohort | Common complete-case cohort | 813 |

**Table S2D. Validation of recomputed variables in the replication cohort**

| **Variable** | **n** | **Published mean** | **Recomputed mean** | **Mean absolute difference** | **Maximum absolute difference** | **Pearson r** |
| --- | --- | --- | --- | --- | --- | --- |
| BMI | 813 | 26.057 | 26.057 | 0.000 | 0.000 | 1.000 |
| TyG | 813 | 8.937 | 8.937 | 0.000 | 0.000 | 1.000 |
| TyG-BMI | 813 | 233.236 | 233.239 | 0.003 | 0.004 | 1.000 |

**Note.** Published and recomputed variables are compared to confirm formula implementation in the replication dataset.

**Table S3A. Spearman correlation coefficients in the primary cohort**

| **Variable** | **TyG** | **TG/HDL-C** | **TG/G ratio** | **CHG** | **SPISE** | **TyG-BMI** | **METS-IR** | **BMI** | **LAD** |
| --- | --- | --- | --- | --- | --- | --- | --- | --- | --- |
| TyG | 1.00 | 0.89 | 0.73 | 0.68 | -0.64 | 0.60 | 0.57 | 0.27 | 0.09 |
| TG/HDL-C | 0.89 | 1.00 | 0.85 | 0.65 | -0.71 | 0.59 | 0.66 | 0.29 | 0.11 |
| TG/G ratio | 0.73 | 0.85 | 1.00 | 0.37 | -0.57 | 0.45 | 0.43 | 0.20 | 0.07 |
| CHG | 0.68 | 0.65 | 0.37 | 1.00 | -0.45 | 0.42 | 0.48 | 0.19 | 0.03 |
| SPISE | -0.64 | -0.71 | -0.57 | -0.45 | 1.00 | -0.97 | -0.97 | -0.86 | -0.30 |
| TyG-BMI | 0.60 | 0.59 | 0.45 | 0.42 | -0.97 | 1.00 | 0.95 | 0.92 | 0.32 |
| METS-IR | 0.57 | 0.66 | 0.43 | 0.48 | -0.97 | 0.95 | 1.00 | 0.87 | 0.31 |
| BMI | 0.27 | 0.29 | 0.20 | 0.19 | -0.86 | 0.92 | 0.87 | 1.00 | 0.35 |
| LAD | 0.09 | 0.11 | 0.07 | 0.03 | -0.30 | 0.32 | 0.31 | 0.35 | 1.00 |

**Table S3B. Spearman correlation P values in the primary cohort**

| **Variable** | **TyG** | **TG/HDL-C** | **TG/G ratio** | **CHG** | **SPISE** | **TyG-BMI** | **METS-IR** | **BMI** | **LAD** |
| --- | --- | --- | --- | --- | --- | --- | --- | --- | --- |
| TyG | — | <0.001 | <0.001 | <0.001 | <0.001 | <0.001 | <0.001 | <0.001 | 0.011 |
| TG/HDL-C | <0.001 | — | <0.001 | <0.001 | <0.001 | <0.001 | <0.001 | <0.001 | 0.002 |
| TG/G ratio | <0.001 | <0.001 | — | <0.001 | <0.001 | <0.001 | <0.001 | <0.001 | 0.062 |
| CHG | <0.001 | <0.001 | <0.001 | — | <0.001 | <0.001 | <0.001 | <0.001 | 0.485 |
| SPISE | <0.001 | <0.001 | <0.001 | <0.001 | — | <0.001 | <0.001 | <0.001 | <0.001 |
| TyG-BMI | <0.001 | <0.001 | <0.001 | <0.001 | <0.001 | — | <0.001 | <0.001 | <0.001 |
| METS-IR | <0.001 | <0.001 | <0.001 | <0.001 | <0.001 | <0.001 | — | <0.001 | <0.001 |
| BMI | <0.001 | <0.001 | <0.001 | <0.001 | <0.001 | <0.001 | <0.001 | — | <0.001 |
| LAD | 0.011 | 0.002 | 0.062 | 0.485 | <0.001 | <0.001 | <0.001 | <0.001 | — |

**Table S3C. Spearman correlation coefficients in the replication cohort**

| **Variable** | **TyG** | **TG/HDL-C** | **TG/G ratio** | **CHG** | **SPISE** | **TyG-BMI** | **METS-IR** | **BMI** | **LAD** |
| --- | --- | --- | --- | --- | --- | --- | --- | --- | --- |
| TyG | 1.00 | 0.82 | 0.59 | 0.66 | -0.55 | 0.54 | 0.47 | 0.20 | -0.08 |
| TG/HDL-C | 0.82 | 1.00 | 0.80 | 0.48 | -0.60 | 0.44 | 0.50 | 0.16 | -0.09 |
| TG/G ratio | 0.59 | 0.80 | 1.00 | 0.09 | -0.43 | 0.27 | 0.21 | 0.06 | -0.14 |
| CHG | 0.66 | 0.48 | 0.09 | 1.00 | -0.39 | 0.43 | 0.46 | 0.21 | 0.01 |
| SPISE | -0.55 | -0.60 | -0.43 | -0.39 | 1.00 | -0.96 | -0.95 | -0.86 | -0.14 |
| TyG-BMI | 0.54 | 0.44 | 0.27 | 0.43 | -0.96 | 1.00 | 0.93 | 0.91 | 0.15 |
| METS-IR | 0.47 | 0.50 | 0.21 | 0.46 | -0.95 | 0.93 | 1.00 | 0.87 | 0.16 |
| BMI | 0.20 | 0.16 | 0.06 | 0.21 | -0.86 | 0.91 | 0.87 | 1.00 | 0.21 |
| LAD | -0.08 | -0.09 | -0.14 | 0.01 | -0.14 | 0.15 | 0.16 | 0.21 | 1.00 |

**Table S3D. Spearman correlation P values in the replication cohort**

| **Variable** | **TyG** | **TG/HDL-C** | **TG/G ratio** | **CHG** | **SPISE** | **TyG-BMI** | **METS-IR** | **BMI** | **LAD** |
| --- | --- | --- | --- | --- | --- | --- | --- | --- | --- |
| TyG | — | <0.001 | <0.001 | <0.001 | <0.001 | <0.001 | <0.001 | <0.001 | 0.029 |
| TG/HDL-C | <0.001 | — | <0.001 | <0.001 | <0.001 | <0.001 | <0.001 | <0.001 | 0.016 |
| TG/G ratio | <0.001 | <0.001 | — | 0.013 | <0.001 | <0.001 | <0.001 | 0.094 | <0.001 |
| CHG | <0.001 | <0.001 | 0.013 | — | <0.001 | <0.001 | <0.001 | <0.001 | 0.689 |
| SPISE | <0.001 | <0.001 | <0.001 | <0.001 | — | <0.001 | <0.001 | <0.001 | <0.001 |
| TyG-BMI | <0.001 | <0.001 | <0.001 | <0.001 | <0.001 | — | <0.001 | <0.001 | <0.001 |
| METS-IR | <0.001 | <0.001 | <0.001 | <0.001 | <0.001 | <0.001 | — | <0.001 | <0.001 |
| BMI | <0.001 | <0.001 | 0.094 | <0.001 | <0.001 | <0.001 | <0.001 | — | <0.001 |
| LAD | 0.029 | 0.016 | <0.001 | 0.689 | <0.001 | <0.001 | <0.001 | <0.001 | — |

**Table S4. Body-size-indexed left atrial diameter sensitivity analyses**

| **Indexing** | **Cohort** | **Marker** | **Formula class** | **n** | **Adjusted difference per 1-SD (95% CI)** | **Nominal P** | **FDR P** | **ΔR², pp** |
| --- | --- | --- | --- | --- | --- | --- | --- | --- |
| LAD/BSA (mm/m²) | Primary cohort | TyG | No BMI in formula | 788 | -0.178 (-0.371, 0.014) | 0.070 | 0.081 | 0.296 |
| LAD/BSA (mm/m²) | Primary cohort | TG/HDL-C | No BMI in formula | 788 | -0.240 (-0.432, -0.048) | 0.015 | 0.025 | 0.537 |
| LAD/BSA (mm/m²) | Primary cohort | TG/G ratio | No BMI in formula | 788 | -0.157 (-0.350, 0.037) | 0.112 | 0.112 | 0.227 |
| LAD/BSA (mm/m²) | Primary cohort | CHG | No BMI in formula | 788 | -0.212 (-0.409, -0.016) | 0.034 | 0.048 | 0.403 |
| LAD/BSA (mm/m²) | Primary cohort | SPISE | BMI algebraically incorporated | 788 | 0.420 (0.226, 0.614) | <0.001 | <0.001 | 1.588 |
| LAD/BSA (mm/m²) | Primary cohort | TyG-BMI | BMI algebraically incorporated | 788 | -0.526 (-0.718, -0.333) | <0.001 | <0.001 | 2.500 |
| LAD/BSA (mm/m²) | Primary cohort | METS-IR | BMI algebraically incorporated | 788 | -0.527 (-0.721, -0.333) | <0.001 | <0.001 | 2.470 |
| LAD/BSA (mm/m²) | Primary cohort | BMI | Prespecified anthropometric benchmark | 788 | -0.547 (-0.740, -0.354) | <0.001 | — | 2.674 |
| LAD/BSA (mm/m²) | Replication cohort | TyG | No BMI in formula | 813 | -0.239 (-0.445, -0.032) | 0.024 | 0.034 | 0.425 |
| LAD/BSA (mm/m²) | Replication cohort | TG/HDL-C | No BMI in formula | 813 | -0.214 (-0.410, -0.017) | 0.033 | 0.034 | 0.376 |
| LAD/BSA (mm/m²) | Replication cohort | TG/G ratio | No BMI in formula | 813 | -0.215 (-0.414, -0.016) | 0.034 | 0.034 | 0.373 |
| LAD/BSA (mm/m²) | Replication cohort | CHG | No BMI in formula | 813 | -0.235 (-0.446, -0.024) | 0.029 | 0.034 | 0.394 |
| LAD/BSA (mm/m²) | Replication cohort | SPISE | BMI algebraically incorporated | 813 | 0.690 (0.498, 0.882) | <0.001 | <0.001 | 3.902 |
| LAD/BSA (mm/m²) | Replication cohort | TyG-BMI | BMI algebraically incorporated | 813 | -0.745 (-0.937, -0.553) | <0.001 | <0.001 | 4.515 |
| LAD/BSA (mm/m²) | Replication cohort | METS-IR | BMI algebraically incorporated | 813 | -0.727 (-0.921, -0.532) | <0.001 | <0.001 | 4.194 |
| LAD/BSA (mm/m²) | Replication cohort | BMI | Prespecified anthropometric benchmark | 813 | -0.758 (-0.947, -0.568) | <0.001 | — | 4.770 |
| LAD/height (mm/m) | Primary cohort | TyG | No BMI in formula | 788 | 0.210 (0.004, 0.416) | 0.045 | 0.079 | 0.364 |
| LAD/height (mm/m) | Primary cohort | TG/HDL-C | No BMI in formula | 788 | 0.117 (-0.090, 0.323) | 0.267 | 0.374 | 0.112 |
| LAD/height (mm/m) | Primary cohort | TG/G ratio | No BMI in formula | 788 | 0.071 (-0.136, 0.278) | 0.501 | 0.565 | 0.041 |
| LAD/height (mm/m) | Primary cohort | CHG | No BMI in formula | 788 | 0.062 (-0.149, 0.272) | 0.565 | 0.565 | 0.030 |
| LAD/height (mm/m) | Primary cohort | SPISE | BMI algebraically incorporated | 788 | -0.834 (-1.036, -0.632) | <0.001 | <0.001 | 5.519 |
| LAD/height (mm/m) | Primary cohort | TyG-BMI | BMI algebraically incorporated | 788 | 0.769 (0.567, 0.972) | <0.001 | <0.001 | 4.722 |
| LAD/height (mm/m) | Primary cohort | METS-IR | BMI algebraically incorporated | 788 | 0.730 (0.525, 0.935) | <0.001 | <0.001 | 4.184 |
| LAD/height (mm/m) | Primary cohort | BMI | Prespecified anthropometric benchmark | 788 | 0.882 (0.681, 1.084) | <0.001 | — | 6.139 |
| LAD/height (mm/m) | Replication cohort | TyG | No BMI in formula | 813 | 0.083 (-0.137, 0.303) | 0.461 | 0.538 | 0.046 |
| LAD/height (mm/m) | Replication cohort | TG/HDL-C | No BMI in formula | 813 | -0.033 (-0.242, 0.177) | 0.759 | 0.759 | 0.008 |
| LAD/height (mm/m) | Replication cohort | TG/G ratio | No BMI in formula | 813 | -0.092 (-0.304, 0.120) | 0.394 | 0.538 | 0.062 |
| LAD/height (mm/m) | Replication cohort | CHG | No BMI in formula | 813 | 0.085 (-0.140, 0.310) | 0.458 | 0.538 | 0.047 |
| LAD/height (mm/m) | Replication cohort | SPISE | BMI algebraically incorporated | 813 | -0.671 (-0.876, -0.466) | <0.001 | <0.001 | 3.319 |
| LAD/height (mm/m) | Replication cohort | TyG-BMI | BMI algebraically incorporated | 813 | 0.669 (0.464, 0.875) | <0.001 | <0.001 | 3.284 |
| LAD/height (mm/m) | Replication cohort | METS-IR | BMI algebraically incorporated | 813 | 0.637 (0.428, 0.846) | <0.001 | <0.001 | 2.903 |
| LAD/height (mm/m) | Replication cohort | BMI | Prespecified anthropometric benchmark | 813 | 0.753 (0.551, 0.955) | <0.001 | — | 4.245 |

**Note.** BSA was calculated using the Mosteller formula. LAD/BSA is expressed in mm/m² and LAD/height in mm/m; the two indexed outcomes are on different physical scales.

**Table S5. Primary-cohort age and AF-duration bounding analyses**

| **Cohort** | **Adjustment** | **Marker** | **n** | **Adjusted LAD difference per 1-SD, mm** | **95% CI low** | **95% CI high** | **Nominal P** | **FDR P** | **ΔR², pp** |
| --- | --- | --- | --- | --- | --- | --- | --- | --- | --- |
| Primary cohort | Harmonized core (without age/AF duration) | TyG | 788 | 0.336 | 0.001 | 0.672 | 0.049 | 0.086 | 0.36 |
| Primary cohort | Harmonized core (without age/AF duration) | TG/HDL-C | 788 | 0.249 | -0.087 | 0.584 | 0.146 | 0.204 | 0.20 |
| Primary cohort | Harmonized core (without age/AF duration) | TG/G ratio | 788 | 0.141 | -0.196 | 0.478 | 0.413 | 0.482 | 0.06 |
| Primary cohort | Harmonized core (without age/AF duration) | CHG | 788 | 0.120 | -0.223 | 0.462 | 0.494 | 0.494 | 0.04 |
| Primary cohort | Harmonized core (without age/AF duration) | SPISE | 788 | -1.350 | -1.679 | -1.021 | <0.001 | <0.001 | 5.66 |
| Primary cohort | Harmonized core (without age/AF duration) | TyG-BMI | 788 | 1.238 | 0.908 | 1.568 | <0.001 | <0.001 | 4.79 |
| Primary cohort | Harmonized core (without age/AF duration) | METS-IR | 788 | 1.203 | 0.870 | 1.537 | <0.001 | <0.001 | 4.45 |
| Primary cohort | Harmonized core (without age/AF duration) | BMI | 788 | 1.421 | 1.092 | 1.749 | <0.001 | — | 6.23 |
| Primary cohort | Core + age | TyG | 788 | 0.382 | 0.051 | 0.712 | 0.024 | 0.041 | 0.47 |
| Primary cohort | Core + age | TG/HDL-C | 788 | 0.335 | 0.003 | 0.666 | 0.048 | 0.067 | 0.36 |
| Primary cohort | Core + age | TG/G ratio | 788 | 0.229 | -0.105 | 0.563 | 0.179 | 0.208 | 0.17 |
| Primary cohort | Core + age | CHG | 788 | 0.162 | -0.176 | 0.501 | 0.346 | 0.346 | 0.08 |
| Primary cohort | Core + age | SPISE | 788 | -1.428 | -1.751 | -1.105 | <0.001 | <0.001 | 6.29 |
| Primary cohort | Core + age | TyG-BMI | 788 | 1.282 | 0.958 | 1.606 | <0.001 | <0.001 | 5.12 |
| Primary cohort | Core + age | METS-IR | 788 | 1.258 | 0.930 | 1.585 | <0.001 | <0.001 | 4.85 |
| Primary cohort | Core + age | BMI | 788 | 1.454 | 1.131 | 1.776 | <0.001 | — | 6.52 |
| Primary cohort | Core + age + AF duration | TyG | 788 | 0.381 | 0.051 | 0.711 | 0.024 | 0.042 | 0.47 |
| Primary cohort | Core + age + AF duration | TG/HDL-C | 788 | 0.341 | 0.009 | 0.672 | 0.044 | 0.062 | 0.37 |
| Primary cohort | Core + age + AF duration | TG/G ratio | 788 | 0.234 | -0.100 | 0.568 | 0.169 | 0.198 | 0.17 |
| Primary cohort | Core + age + AF duration | CHG | 788 | 0.162 | -0.176 | 0.500 | 0.346 | 0.346 | 0.08 |
| Primary cohort | Core + age + AF duration | SPISE | 788 | -1.424 | -1.747 | -1.101 | <0.001 | <0.001 | 6.26 |
| Primary cohort | Core + age + AF duration | TyG-BMI | 788 | 1.278 | 0.953 | 1.602 | <0.001 | <0.001 | 5.08 |
| Primary cohort | Core + age + AF duration | METS-IR | 788 | 1.255 | 0.928 | 1.583 | <0.001 | <0.001 | 4.83 |
| Primary cohort | Core + age + AF duration | BMI | 788 | 1.448 | 1.126 | 1.771 | <0.001 | — | 6.46 |

**Note.** This analysis quantifies how the primary-cohort estimates change when age and AF duration are added to the harmonized core adjustment set.

**Table S6. Reduced and minimally adjusted sensitivity analyses**

| **Cohort** | **Adjustment** | **Marker** | **n** | **Adjusted LAD difference per 1-SD, mm** | **95% CI low** | **95% CI high** | **Nominal P** | **FDR P** | **ΔR², pp** |
| --- | --- | --- | --- | --- | --- | --- | --- | --- | --- |
| Primary cohort | Sex only | TyG | 788 | 0.404 | 0.017 | 0.791 | 0.041 | 0.071 | 0.53 |
| Primary cohort | Sex only | TG/HDL-C | 788 | 0.231 | -0.158 | 0.619 | 0.244 | 0.341 | 0.17 |
| Primary cohort | Sex only | TG/G ratio | 788 | 0.026 | -0.361 | 0.414 | 0.893 | 0.893 | <0.01 |
| Primary cohort | Sex only | CHG | 788 | 0.103 | -0.286 | 0.491 | 0.604 | 0.705 | 0.03 |
| Primary cohort | Sex only | SPISE | 788 | -1.675 | -2.045 | -1.304 | <0.001 | <0.001 | 9.11 |
| Primary cohort | Sex only | TyG-BMI | 788 | 1.605 | 1.235 | 1.976 | <0.001 | <0.001 | 8.41 |
| Primary cohort | Sex only | METS-IR | 788 | 1.565 | 1.190 | 1.939 | <0.001 | <0.001 | 7.88 |
| Primary cohort | Sex only | BMI | 788 | 1.841 | 1.475 | 2.206 | <0.001 | — | 11.04 |
| Primary cohort | Age + sex | TyG | 788 | 0.444 | 0.063 | 0.825 | 0.022 | 0.039 | 0.64 |
| Primary cohort | Age + sex | TG/HDL-C | 788 | 0.329 | -0.055 | 0.713 | 0.093 | 0.131 | 0.35 |
| Primary cohort | Age + sex | TG/G ratio | 788 | 0.139 | -0.245 | 0.523 | 0.478 | 0.478 | 0.06 |
| Primary cohort | Age + sex | CHG | 788 | 0.168 | -0.215 | 0.551 | 0.390 | 0.455 | 0.09 |
| Primary cohort | Age + sex | SPISE | 788 | -1.724 | -2.087 | -1.361 | <0.001 | <0.001 | 9.63 |
| Primary cohort | Age + sex | TyG-BMI | 788 | 1.622 | 1.257 | 1.986 | <0.001 | <0.001 | 8.57 |
| Primary cohort | Age + sex | METS-IR | 788 | 1.588 | 1.220 | 1.955 | <0.001 | <0.001 | 8.10 |
| Primary cohort | Age + sex | BMI | 788 | 1.843 | 1.484 | 2.202 | <0.001 | — | 11.06 |
| Primary cohort | Age + sex + HTN + DM + CAD + stroke | TyG | 788 | 0.405 | 0.024 | 0.785 | 0.037 | 0.065 | 0.53 |
| Primary cohort | Age + sex + HTN + DM + CAD + stroke | TG/HDL-C | 788 | 0.316 | -0.066 | 0.698 | 0.105 | 0.147 | 0.32 |
| Primary cohort | Age + sex + HTN + DM + CAD + stroke | TG/G ratio | 788 | 0.168 | -0.216 | 0.553 | 0.391 | 0.391 | 0.09 |
| Primary cohort | Age + sex + HTN + DM + CAD + stroke | CHG | 788 | 0.197 | -0.192 | 0.586 | 0.321 | 0.374 | 0.12 |
| Primary cohort | Age + sex + HTN + DM + CAD + stroke | SPISE | 788 | -1.694 | -2.062 | -1.325 | <0.001 | <0.001 | 8.96 |
| Primary cohort | Age + sex + HTN + DM + CAD + stroke | TyG-BMI | 788 | 1.584 | 1.216 | 1.952 | <0.001 | <0.001 | 7.94 |
| Primary cohort | Age + sex + HTN + DM + CAD + stroke | METS-IR | 788 | 1.547 | 1.174 | 1.920 | <0.001 | <0.001 | 7.43 |
| Primary cohort | Age + sex + HTN + DM + CAD + stroke | BMI | 788 | 1.816 | 1.452 | 2.179 | <0.001 | — | 10.39 |
| Primary cohort | Harmonized core | TyG | 788 | 0.336 | 0.001 | 0.672 | 0.049 | 0.086 | 0.36 |
| Primary cohort | Harmonized core | TG/HDL-C | 788 | 0.249 | -0.087 | 0.584 | 0.146 | 0.204 | 0.20 |
| Primary cohort | Harmonized core | TG/G ratio | 788 | 0.141 | -0.196 | 0.478 | 0.413 | 0.482 | 0.06 |
| Primary cohort | Harmonized core | CHG | 788 | 0.120 | -0.223 | 0.462 | 0.494 | 0.494 | 0.04 |
| Primary cohort | Harmonized core | SPISE | 788 | -1.350 | -1.679 | -1.021 | <0.001 | <0.001 | 5.66 |
| Primary cohort | Harmonized core | TyG-BMI | 788 | 1.238 | 0.908 | 1.568 | <0.001 | <0.001 | 4.79 |
| Primary cohort | Harmonized core | METS-IR | 788 | 1.203 | 0.870 | 1.537 | <0.001 | <0.001 | 4.45 |
| Primary cohort | Harmonized core | BMI | 788 | 1.421 | 1.092 | 1.749 | <0.001 | — | 6.23 |
| Replication cohort | Sex only | TyG | 813 | -0.544 | -0.956 | -0.131 | 0.010 | 0.014 | 0.80 |
| Replication cohort | Sex only | TG/HDL-C | 813 | -0.375 | -0.791 | 0.041 | 0.078 | 0.090 | 0.37 |
| Replication cohort | Sex only | TG/G ratio | 813 | -0.613 | -1.027 | -0.200 | 0.004 | 0.006 | 1.01 |
| Replication cohort | Sex only | CHG | 813 | -0.002 | -0.418 | 0.413 | 0.991 | 0.991 | <0.01 |
| Replication cohort | Sex only | SPISE | 813 | -0.750 | -1.164 | -0.337 | <0.001 | <0.001 | 1.50 |
| Replication cohort | Sex only | TyG-BMI | 813 | 0.890 | 0.479 | 1.300 | <0.001 | <0.001 | 2.13 |
| Replication cohort | Sex only | METS-IR | 813 | 0.913 | 0.500 | 1.326 | <0.001 | <0.001 | 2.21 |
| Replication cohort | Sex only | BMI | 813 | 1.323 | 0.918 | 1.728 | <0.001 | — | 4.71 |
| Replication cohort | Sex + HTN + DM + CAD + stroke | TyG | 813 | -0.577 | -1.004 | -0.150 | 0.008 | 0.014 | 0.83 |
| Replication cohort | Sex + HTN + DM + CAD + stroke | TG/HDL-C | 813 | -0.322 | -0.742 | 0.097 | 0.132 | 0.154 | 0.27 |
| Replication cohort | Sex + HTN + DM + CAD + stroke | TG/G ratio | 813 | -0.540 | -0.962 | -0.119 | 0.012 | 0.017 | 0.75 |
| Replication cohort | Sex + HTN + DM + CAD + stroke | CHG | 813 | -0.060 | -0.510 | 0.389 | 0.792 | 0.792 | <0.01 |
| Replication cohort | Sex + HTN + DM + CAD + stroke | SPISE | 813 | -0.737 | -1.154 | -0.320 | <0.001 | 0.001 | 1.42 |
| Replication cohort | Sex + HTN + DM + CAD + stroke | TyG-BMI | 813 | 0.872 | 0.453 | 1.291 | <0.001 | <0.001 | 1.95 |
| Replication cohort | Sex + HTN + DM + CAD + stroke | METS-IR | 813 | 0.900 | 0.476 | 1.325 | <0.001 | <0.001 | 2.03 |
| Replication cohort | Sex + HTN + DM + CAD + stroke | BMI | 813 | 1.296 | 0.885 | 1.707 | <0.001 | — | 4.38 |
| Replication cohort | Harmonized core | TyG | 813 | 0.225 | -0.142 | 0.592 | 0.230 | 0.321 | 0.12 |
| Replication cohort | Harmonized core | TG/HDL-C | 813 | 0.026 | -0.324 | 0.376 | 0.885 | 0.885 | <0.01 |
| Replication cohort | Harmonized core | TG/G ratio | 813 | -0.112 | -0.466 | 0.241 | 0.532 | 0.621 | 0.03 |
| Replication cohort | Harmonized core | CHG | 813 | 0.282 | -0.093 | 0.657 | 0.140 | 0.246 | 0.18 |
| Replication cohort | Harmonized core | SPISE | 813 | -1.179 | -1.520 | -0.838 | <0.001 | <0.001 | 3.57 |
| Replication cohort | Harmonized core | TyG-BMI | 813 | 1.174 | 0.831 | 1.516 | <0.001 | <0.001 | 3.51 |
| Replication cohort | Harmonized core | METS-IR | 813 | 1.160 | 0.813 | 1.507 | <0.001 | <0.001 | 3.35 |
| Replication cohort | Harmonized core | BMI | 813 | 1.284 | 0.947 | 1.621 | <0.001 | — | 4.29 |

**Note.** Reduced adjustment sets assess sensitivity to covariate specification, including concern that AF phenotype and LVEF may be downstream variables. No causal effect is claimed.

**Table S7A. Collinearity diagnostics for BMI-index joint models**

| **Cohort** | **Index** | **n** | **BMI VIF** | **BMI tolerance** | **Index VIF** | **Index tolerance** |
| --- | --- | --- | --- | --- | --- | --- |
| Primary cohort | SPISE | 788 | 3.231 | 0.309 | 3.210 | 0.312 |
| Primary cohort | TyG-BMI | 788 | 7.849 | 0.127 | 7.757 | 0.129 |
| Primary cohort | METS-IR | 788 | 5.214 | 0.192 | 5.235 | 0.191 |
| Replication cohort | SPISE | 813 | 3.916 | 0.255 | 3.974 | 0.252 |
| Replication cohort | TyG-BMI | 813 | 7.233 | 0.138 | 7.392 | 0.135 |
| Replication cohort | METS-IR | 813 | 4.503 | 0.222 | 4.715 | 0.212 |

**Note.** VIF/tolerance quantify collinearity in models containing clinical covariates, BMI, and the specified index.

**Table S7B. Descriptive coefficients from joint BMI-index models**

| **Cohort** | **Index** | **n** | **BMI coefficient** | **BMI 95% CI low** | **BMI 95% CI high** | **BMI P** | **Index coefficient** | **Index 95% CI low** | **Index 95% CI high** | **Index P** | **Index FDR P** |
| --- | --- | --- | --- | --- | --- | --- | --- | --- | --- | --- | --- |
| Primary cohort | SPISE | 788 | 0.947 | 0.374 | 1.520 | 0.001 | -0.575 | -1.147 | -0.004 | 0.048 | 0.145 |
| Primary cohort | TyG-BMI | 788 | 1.947 | 1.052 | 2.842 | <0.001 | -0.562 | -1.451 | 0.327 | 0.215 | 0.323 |
| Primary cohort | METS-IR | 788 | 1.720 | 0.990 | 2.449 | <0.001 | -0.335 | -1.066 | 0.396 | 0.368 | 0.368 |
| Replication cohort | SPISE | 813 | 1.060 | 0.405 | 1.715 | 0.002 | -0.263 | -0.923 | 0.396 | 0.434 | 0.928 |
| Replication cohort | TyG-BMI | 813 | 1.462 | 0.571 | 2.352 | 0.001 | -0.194 | -1.094 | 0.706 | 0.672 | 0.928 |
| Replication cohort | METS-IR | 813 | 1.256 | 0.553 | 1.958 | <0.001 | 0.033 | -0.686 | 0.752 | 0.928 | 0.928 |

**Note.** Joint BMI-index coefficients are retained for transparency but are treated as descriptive rather than as evidence of separable biological effects because of collinearity. Coefficients are adjusted LAD differences per 1-SD increase, in mm.

**Table S8. Restricted cubic-spline tests**

| **Cohort** | **Index** | **P overall** | **P nonlinearity** | **Knot 1 (Z)** | **Knot 2 (Z)** | **Knot 3 (Z)** | **Knot 4 (Z)** |
| --- | --- | --- | --- | --- | --- | --- | --- |
| Primary cohort | BMI | <0.001 | 0.002 | -1.49 | -0.38 | 0.24 | 1.64 |
| Primary cohort | SPISE | <0.001 | 0.897 | -1.40 | -0.47 | 0.22 | 1.78 |
| Primary cohort | TyG-BMI | <0.001 | 0.002 | -1.39 | -0.37 | 0.26 | 1.51 |
| Primary cohort | METS-IR | <0.001 | 0.002 | -1.43 | -0.39 | 0.29 | 1.60 |
| Replication cohort | BMI | <0.001 | 0.031 | -1.53 | -0.37 | 0.32 | 1.73 |
| Replication cohort | SPISE | <0.001 | 0.127 | -1.45 | -0.42 | 0.20 | 1.83 |
| Replication cohort | TyG-BMI | <0.001 | 0.003 | -1.52 | -0.37 | 0.31 | 1.71 |
| Replication cohort | METS-IR | <0.001 | <0.001 | -1.49 | -0.39 | 0.32 | 1.70 |

**Note.** Overall and nonlinearity P values correspond to the restricted cubic-spline analyses shown in Supplementary Figure S2.

**Table S9. Exploratory AF-phenotype interaction analyses**

| **Panel** | **Cohort** | **Adjustment** | **Index** | **Difference in LAD slope (persistent - paroxysmal), mm** | **95% CI low** | **95% CI high** | **P for interaction** | **FDR P** |
| --- | --- | --- | --- | --- | --- | --- | --- | --- |
| A. Harmonized cross-cohort comparison | Primary cohort | Harmonized core | TyG | 0.187 | -0.491 | 0.866 | 0.588 | 0.619 |
| A. Harmonized cross-cohort comparison | Primary cohort | Harmonized core | TG/HDL-C | 0.541 | -0.148 | 1.230 | 0.124 | 0.215 |
| A. Harmonized cross-cohort comparison | Primary cohort | Harmonized core | TG/G ratio | 0.646 | -0.076 | 1.367 | 0.080 | 0.186 |
| A. Harmonized cross-cohort comparison | Primary cohort | Harmonized core | CHG | -0.174 | -0.860 | 0.513 | 0.619 | 0.619 |
| A. Harmonized cross-cohort comparison | Primary cohort | Harmonized core | SPISE | -0.479 | -1.137 | 0.179 | 0.154 | 0.215 |
| A. Harmonized cross-cohort comparison | Primary cohort | Harmonized core | TyG-BMI | 0.744 | 0.075 | 1.413 | 0.029 | 0.103 |
| A. Harmonized cross-cohort comparison | Primary cohort | Harmonized core | METS-IR | 0.762 | 0.090 | 1.434 | 0.026 | 0.103 |
| A. Harmonized cross-cohort comparison | Replication cohort | Harmonized core | TyG | -0.404 | -1.119 | 0.310 | 0.267 | 0.353 |
| A. Harmonized cross-cohort comparison | Replication cohort | Harmonized core | TG/HDL-C | -0.529 | -1.230 | 0.171 | 0.138 | 0.353 |
| A. Harmonized cross-cohort comparison | Replication cohort | Harmonized core | TG/G ratio | -0.365 | -1.058 | 0.329 | 0.303 | 0.353 |
| A. Harmonized cross-cohort comparison | Replication cohort | Harmonized core | CHG | 0.032 | -0.662 | 0.725 | 0.929 | 0.929 |
| A. Harmonized cross-cohort comparison | Replication cohort | Harmonized core | SPISE | 0.590 | -0.097 | 1.278 | 0.092 | 0.353 |
| A. Harmonized cross-cohort comparison | Replication cohort | Harmonized core | TyG-BMI | -0.403 | -1.075 | 0.269 | 0.240 | 0.353 |
| A. Harmonized cross-cohort comparison | Replication cohort | Harmonized core | METS-IR | -0.429 | -1.100 | 0.242 | 0.210 | 0.353 |
| B. Primary age/AF-duration-adjusted sensitivity | Primary cohort | Core + age + AF duration | TyG | 0.202 | -0.468 | 0.871 | 0.554 | 0.647 |
| B. Primary age/AF-duration-adjusted sensitivity | Primary cohort | Core + age + AF duration | TG/HDL-C | 0.547 | -0.132 | 1.225 | 0.114 | 0.172 |
| B. Primary age/AF-duration-adjusted sensitivity | Primary cohort | Core + age + AF duration | TG/G ratio | 0.629 | -0.082 | 1.340 | 0.083 | 0.172 |
| B. Primary age/AF-duration-adjusted sensitivity | Primary cohort | Core + age + AF duration | CHG | -0.142 | -0.820 | 0.536 | 0.681 | 0.681 |
| B. Primary age/AF-duration-adjusted sensitivity | Primary cohort | Core + age + AF duration | SPISE | -0.507 | -1.152 | 0.137 | 0.123 | 0.172 |
| B. Primary age/AF-duration-adjusted sensitivity | Primary cohort | Core + age + AF duration | TyG-BMI | 0.817 | 0.161 | 1.474 | 0.015 | 0.052 |
| B. Primary age/AF-duration-adjusted sensitivity | Primary cohort | Core + age + AF duration | METS-IR | 0.830 | 0.171 | 1.490 | 0.014 | 0.052 |

**Note.** No interaction met the prespecified BH-FDR threshold in either cohort.

**Table S10. Quantile regression across the LAD distribution**

| **Index** | **Conditional LAD quantile** | **Adjusted LAD difference per 1-SD, mm** | **95% CI low** | **95% CI high** | **P** | **FDR P within quantile** |
| --- | --- | --- | --- | --- | --- | --- |
| TyG | 0.250 | 0.499 | 0.127 | 0.870 | 0.009 | 0.015 |
| TyG | 0.500 | 0.572 | 0.204 | 0.939 | 0.002 | 0.003 |
| TyG | 0.750 | 0.356 | -0.066 | 0.777 | 0.099 | 0.115 |
| TyG | 0.900 | 0.106 | -0.225 | 0.438 | 0.529 | 0.835 |
| TG/HDL-C | 0.250 | 0.470 | 0.105 | 0.836 | 0.012 | 0.017 |
| TG/HDL-C | 0.500 | 0.425 | 0.107 | 0.743 | 0.009 | 0.010 |
| TG/HDL-C | 0.750 | 0.270 | 0.078 | 0.462 | 0.006 | 0.010 |
| TG/HDL-C | 0.900 | 0.131 | -0.354 | 0.616 | 0.596 | 0.835 |
| TG/G ratio | 0.250 | 0.253 | 0.015 | 0.492 | 0.038 | 0.044 |
| TG/G ratio | 0.500 | 0.133 | 0.076 | 0.189 | <0.001 | <0.001 |
| TG/G ratio | 0.750 | 0.179 | -0.025 | 0.382 | 0.086 | 0.115 |
| TG/G ratio | 0.900 | -0.069 | -0.732 | 0.594 | 0.838 | 0.838 |
| CHG | 0.250 | 0.265 | -0.082 | 0.613 | 0.135 | 0.135 |
| CHG | 0.500 | 0.249 | -0.138 | 0.635 | 0.207 | 0.207 |
| CHG | 0.750 | 0.182 | -0.240 | 0.604 | 0.398 | 0.398 |
| CHG | 0.900 | 0.064 | -0.429 | 0.558 | 0.798 | 0.838 |
| SPISE | 0.250 | -1.484 | -1.859 | -1.109 | <0.001 | <0.001 |
| SPISE | 0.500 | -1.457 | -1.802 | -1.113 | <0.001 | <0.001 |
| SPISE | 0.750 | -1.576 | -2.029 | -1.123 | <0.001 | <0.001 |
| SPISE | 0.900 | -1.387 | -1.934 | -0.841 | <0.001 | <0.001 |
| TyG-BMI | 0.250 | 1.470 | 1.092 | 1.847 | <0.001 | <0.001 |
| TyG-BMI | 0.500 | 1.428 | 1.023 | 1.832 | <0.001 | <0.001 |
| TyG-BMI | 0.750 | 1.708 | 1.483 | 1.934 | <0.001 | <0.001 |
| TyG-BMI | 0.900 | 1.621 | 1.071 | 2.171 | <0.001 | <0.001 |
| METS-IR | 0.250 | 1.525 | 1.107 | 1.942 | <0.001 | <0.001 |
| METS-IR | 0.500 | 1.518 | 1.107 | 1.929 | <0.001 | <0.001 |
| METS-IR | 0.750 | 1.615 | 1.148 | 2.082 | <0.001 | <0.001 |
| METS-IR | 0.900 | 1.601 | 1.080 | 2.122 | <0.001 | <0.001 |

**Table S11A. Secondary diameter-defined LAE association and discrimination analyses**

| **Cohort** | **Index** | **n** | **OR** | **95% CI low** | **95% CI high** | **P** | **FDR P** | **AUC** | **AUC 95% CI low** | **AUC 95% CI high** | **ΔAUC vs clinical** | **DeLong P vs clinical** | **Brier** |
| --- | --- | --- | --- | --- | --- | --- | --- | --- | --- | --- | --- | --- | --- |
| Primary cohort | SPISE | 788 | 0.602 | 0.503 | 0.715 | <0.001 | <0.001 | 0.794 | 0.763 | 0.825 | 0.024 | 0.006 | 0.181 |
| Primary cohort | TyG-BMI | 788 | 1.653 | 1.367 | 2.016 | <0.001 | <0.001 | 0.795 | 0.764 | 0.826 | 0.025 | 0.003 | 0.182 |
| Primary cohort | METS-IR | 788 | 1.613 | 1.336 | 1.963 | <0.001 | <0.001 | 0.791 | 0.760 | 0.823 | 0.022 | 0.010 | 0.183 |
| Primary cohort | BMI | 788 | 1.692 | 1.398 | 2.065 | <0.001 | — | 0.797 | 0.766 | 0.828 | 0.027 | 0.002 | 0.181 |
| Replication cohort | SPISE | 813 | 0.678 | 0.569 | 0.805 | <0.001 | <0.001 | 0.815 | 0.786 | 0.844 | 0.012 | 0.015 | 0.175 |
| Replication cohort | TyG-BMI | 813 | 1.389 | 1.171 | 1.652 | <0.001 | <0.001 | 0.813 | 0.783 | 0.842 | 0.010 | 0.027 | 0.176 |
| Replication cohort | METS-IR | 813 | 1.319 | 1.110 | 1.571 | 0.002 | 0.004 | 0.810 | 0.780 | 0.839 | 0.007 | 0.075 | 0.177 |
| Replication cohort | BMI | 813 | 1.383 | 1.168 | 1.643 | <0.001 | — | 0.812 | 0.782 | 0.841 | 0.009 | 0.034 | 0.176 |

**Note.** Diameter-defined LAE is a secondary dichotomized representation of LAD and is not an independent validation endpoint or prospective decision outcome.

**Table S11B. Bootstrap optimism-corrected performance for secondary LAE models**

| **Cohort** | **Adjustment** | **n** | **Model** | **Apparent AUC** | **Optimism-corrected AUC** | **Apparent Brier score** | **Optimism-corrected Brier score** | **Optimism-corrected calibration slope** | **Optimism-corrected calibration intercept** | **Valid bootstraps** |
| --- | --- | --- | --- | --- | --- | --- | --- | --- | --- | --- |
| Primary cohort | Harmonized core | 788 | Clinical model | 0.770 | 0.761 | 0.190 | 0.194 | 0.957 | -0.004 | 1000 |
| Primary cohort | Harmonized core | 788 | Clinical + BMI | 0.797 | 0.788 | 0.181 | 0.185 | 0.951 | 0.001 | 1000 |
| Primary cohort | Harmonized core | 788 | Clinical + SPISE | 0.794 | 0.786 | 0.181 | 0.186 | 0.954 | 0.001 | 1000 |
| Primary cohort | Harmonized core | 788 | Clinical + TyG-BMI | 0.795 | 0.786 | 0.182 | 0.186 | 0.949 | 0.002 | 1000 |
| Primary cohort | Harmonized core | 788 | Clinical + METS-IR | 0.791 | 0.782 | 0.183 | 0.188 | 0.948 | -0.003 | 1000 |
| Replication cohort | Harmonized core | 813 | Clinical model | 0.803 | 0.798 | 0.179 | 0.182 | 0.972 | -0.001 | 1000 |
| Replication cohort | Harmonized core | 813 | Clinical + BMI | 0.812 | 0.805 | 0.176 | 0.180 | 0.960 | -0.001 | 1000 |
| Replication cohort | Harmonized core | 813 | Clinical + SPISE | 0.815 | 0.809 | 0.175 | 0.179 | 0.966 | -0.004 | 1000 |
| Replication cohort | Harmonized core | 813 | Clinical + TyG-BMI | 0.813 | 0.805 | 0.176 | 0.180 | 0.962 | 0.004 | 1000 |
| Replication cohort | Harmonized core | 813 | Clinical + METS-IR | 0.810 | 0.803 | 0.177 | 0.181 | 0.965 | -0.005 | 1000 |

**Note.** Diameter-defined LAE is a secondary dichotomized representation of LAD and is not an independent validation endpoint or prospective decision outcome.

**Table S12. Bootstrap ranking stability for |β|, ΔR², and ΔAUC**

| **Cohort** | **Index** | **Rank-1 probability (%) \|β\|** | **Rank-1 probability (%) ΔR²** | **Rank-1 probability (%) ΔAUC** | **Median rank \|β\|** | **Median rank ΔR²** | **Median rank ΔAUC** | **Valid bootstraps** |
| --- | --- | --- | --- | --- | --- | --- | --- | --- |
| Primary cohort | CHG | 0.0 | 0.0 | 0.1 | 6.000 | 6.000 | 6.000 | 1000.0 |
| Primary cohort | METS-IR | 4.2 | 3.3 | 5.1 | 2.000 | 3.000 | 3.000 | 1000.0 |
| Primary cohort | SPISE | 62.8 | 65.2 | 44.9 | 1.000 | 1.000 | 2.000 | 1000.0 |
| Primary cohort | TG/G ratio | 0.0 | 0.0 | 0.0 | 6.000 | 6.000 | 6.000 | 1000.0 |
| Primary cohort | TG/HDL-C | 0.0 | 0.0 | 0.0 | 5.000 | 5.000 | 5.000 | 1000.0 |
| Primary cohort | TyG-BMI | 33.0 | 31.5 | 49.9 | 2.000 | 2.000 | 2.000 | 1000.0 |
| Primary cohort | TyG | 0.0 | 0.0 | 0.0 | 4.000 | 4.000 | 4.000 | 1000.0 |
| Replication cohort | CHG | 0.0 | 0.0 | 0.1 | 5.000 | 5.000 | 6.000 | 1000.0 |
| Replication cohort | METS-IR | 26.4 | 17.4 | 0.0 | 2.000 | 3.000 | 3.000 | 1000.0 |
| Replication cohort | SPISE | 40.4 | 46.6 | 93.9 | 2.000 | 2.000 | 1.000 | 1000.0 |
| Replication cohort | TG/G ratio | 0.0 | 0.0 | 0.1 | 6.000 | 6.000 | 6.000 | 1000.0 |
| Replication cohort | TG/HDL-C | 0.0 | 0.0 | 0.1 | 6.000 | 6.000 | 6.000 | 1000.0 |
| Replication cohort | TyG-BMI | 33.2 | 36.0 | 4.8 | 2.000 | 2.000 | 2.000 | 1000.0 |
| Replication cohort | TyG | 0.0 | 0.0 | 1.0 | 5.000 | 5.000 | 4.000 | 1000.0 |

**Note.** Rank-1 probabilities were estimated by bootstrap resampling. ΔR² refers to incremental R² beyond the harmonized clinical model; ΔAUC refers to the secondary diameter-defined LAE analysis.

**Table S13. Other sensitivity analyses**

| **Cohort** | **Model role** | **Specification** | **Method** | **Marker** | **n** | **Adjusted LAD difference per 1-SD** | **95% CI low** | **95% CI high** | **P** | **FDR P** | **ΔR², pp** |
| --- | --- | --- | --- | --- | --- | --- | --- | --- | --- | --- | --- |
| Primary cohort | Sensitivity analysis | Participants without diabetes | Conventional SE | TyG | 664 | 0.266 | -0.101 | 0.633 | 0.155 | 0.271 | 0.221 |
| Primary cohort | Sensitivity analysis | Participants without diabetes | Conventional SE | TG/HDL-C | 664 | 0.115 | -0.251 | 0.480 | 0.538 | 0.628 | 0.041 |
| Primary cohort | Sensitivity analysis | Participants without diabetes | Conventional SE | TG/G ratio | 664 | 0.019 | -0.333 | 0.371 | 0.916 | 0.916 | 0.001 |
| Primary cohort | Sensitivity analysis | Participants without diabetes | Conventional SE | CHG | 664 | 0.123 | -0.268 | 0.514 | 0.536 | 0.628 | 0.042 |
| Primary cohort | Sensitivity analysis | Participants without diabetes | Conventional SE | SPISE | 664 | -1.455 | -1.816 | -1.094 | <0.001 | <0.001 | 6.255 |
| Primary cohort | Sensitivity analysis | Participants without diabetes | Conventional SE | TyG-BMI | 664 | 1.319 | 0.959 | 1.680 | <0.001 | <0.001 | 5.235 |
| Primary cohort | Sensitivity analysis | Participants without diabetes | Conventional SE | METS-IR | 664 | 1.288 | 0.924 | 1.652 | <0.001 | <0.001 | 4.900 |
| Primary cohort | Sensitivity analysis | Participants without diabetes | Conventional SE | BMI | 664 | 1.567 | 1.210 | 1.924 | <0.001 | — | 7.284 |
| Primary cohort | Sensitivity analysis | HC3 robust standard errors | HC3 | TyG | 788 | 0.336 | 0.010 | 0.663 | 0.044 | 0.077 | 0.365 |
| Primary cohort | Sensitivity analysis | HC3 robust standard errors | HC3 | TG/HDL-C | 788 | 0.249 | -0.070 | 0.568 | 0.127 | 0.178 | 0.200 |
| Primary cohort | Sensitivity analysis | HC3 robust standard errors | HC3 | TG/G ratio | 788 | 0.141 | -0.161 | 0.443 | 0.361 | 0.421 | 0.063 |
| Primary cohort | Sensitivity analysis | HC3 robust standard errors | HC3 | CHG | 788 | 0.120 | -0.215 | 0.454 | 0.484 | 0.484 | 0.044 |
| Primary cohort | Sensitivity analysis | HC3 robust standard errors | HC3 | SPISE | 788 | -1.350 | -1.682 | -1.018 | <0.001 | <0.001 | 5.664 |
| Primary cohort | Sensitivity analysis | HC3 robust standard errors | HC3 | TyG-BMI | 788 | 1.238 | 0.476 | 2.001 | 0.002 | 0.004 | 4.791 |
| Primary cohort | Sensitivity analysis | HC3 robust standard errors | HC3 | METS-IR | 788 | 1.203 | 0.505 | 1.902 | <0.001 | 0.003 | 4.453 |
| Primary cohort | Sensitivity analysis | HC3 robust standard errors | HC3 | BMI | 788 | 1.421 | 0.723 | 2.119 | <0.001 | — | 6.233 |
| Replication cohort | Reference model | Harmonized core | Conventional SE | TyG | 813 | 0.225 | -0.142 | 0.592 | 0.230 | 0.321 | 0.118 |
| Replication cohort | Reference model | Harmonized core | Conventional SE | TG/HDL-C | 813 | 0.026 | -0.324 | 0.376 | 0.885 | 0.885 | 0.002 |
| Replication cohort | Reference model | Harmonized core | Conventional SE | TG/G ratio | 813 | -0.112 | -0.466 | 0.241 | 0.532 | 0.621 | 0.032 |
| Replication cohort | Reference model | Harmonized core | Conventional SE | CHG | 813 | 0.282 | -0.093 | 0.657 | 0.140 | 0.246 | 0.178 |
| Replication cohort | Reference model | Harmonized core | Conventional SE | SPISE | 813 | -1.179 | -1.520 | -0.838 | <0.001 | <0.001 | 3.567 |
| Replication cohort | Reference model | Harmonized core | Conventional SE | TyG-BMI | 813 | 1.174 | 0.831 | 1.516 | <0.001 | <0.001 | 3.510 |
| Replication cohort | Reference model | Harmonized core | Conventional SE | METS-IR | 813 | 1.160 | 0.813 | 1.507 | <0.001 | <0.001 | 3.349 |
| Replication cohort | Reference model | Harmonized core | Conventional SE | BMI | 813 | 1.284 | 0.947 | 1.621 | <0.001 | — | 4.294 |
| Replication cohort | Sensitivity analysis | Core + smoking + drinking | Conventional SE | TyG | 813 | 0.176 | -0.194 | 0.546 | 0.350 | 0.476 | 0.071 |
| Replication cohort | Sensitivity analysis | Core + smoking + drinking | Conventional SE | TG/HDL-C | 813 | -0.011 | -0.362 | 0.340 | 0.951 | 0.951 | <0.001 |
| Replication cohort | Sensitivity analysis | Core + smoking + drinking | Conventional SE | TG/G ratio | 813 | -0.149 | -0.504 | 0.205 | 0.408 | 0.476 | 0.056 |
| Replication cohort | Sensitivity analysis | Core + smoking + drinking | Conventional SE | CHG | 813 | 0.248 | -0.129 | 0.625 | 0.197 | 0.345 | 0.136 |
| Replication cohort | Sensitivity analysis | Core + smoking + drinking | Conventional SE | SPISE | 813 | -1.168 | -1.523 | -0.812 | <0.001 | <0.001 | 3.229 |
| Replication cohort | Sensitivity analysis | Core + smoking + drinking | Conventional SE | TyG-BMI | 813 | 1.159 | 0.803 | 1.515 | <0.001 | <0.001 | 3.179 |
| Replication cohort | Sensitivity analysis | Core + smoking + drinking | Conventional SE | METS-IR | 813 | 1.140 | 0.781 | 1.499 | <0.001 | <0.001 | 3.028 |
| Replication cohort | Sensitivity analysis | Core + smoking + drinking | Conventional SE | BMI | 813 | 1.280 | 0.930 | 1.629 | <0.001 | — | 3.971 |

**Note.** In the replication cohort, the harmonized core specification is the reference model and core + smoking + drinking is the sensitivity model. HC3 changes standard errors and confidence intervals but does not change fitted coefficients or R².

**Table S14. Per-IQR exposure scaling**

| **Cohort** | **Marker** | **Formula class** | **IQR** | **Adjusted LAD difference per IQR, mm** | **95% CI low** | **95% CI high** | **P** | **FDR P** | **ΔR², pp** |
| --- | --- | --- | --- | --- | --- | --- | --- | --- | --- |
| Primary cohort | TyG | No BMI in formula | 0.672 | 0.438 | 0.002 | 0.874 | 0.049 | 0.086 | 0.365 |
| Primary cohort | TG/HDL-C | No BMI in formula | 2.417 | 0.239 | -0.083 | 0.562 | 0.146 | 0.204 | 0.200 |
| Primary cohort | TG/G ratio | No BMI in formula | 0.754 | 0.112 | -0.156 | 0.379 | 0.413 | 0.482 | 0.063 |
| Primary cohort | CHG | No BMI in formula | 0.475 | 0.164 | -0.306 | 0.634 | 0.494 | 0.494 | 0.044 |
| Primary cohort | SPISE | BMI algebraically incorporated | 1.912 | -1.732 | -2.153 | -1.310 | <0.001 | <0.001 | 5.664 |
| Primary cohort | TyG-BMI | BMI algebraically incorporated | 43.935 | 1.539 | 1.129 | 1.949 | <0.001 | <0.001 | 4.791 |
| Primary cohort | METS-IR | BMI algebraically incorporated | 8.835 | 1.504 | 1.087 | 1.920 | <0.001 | <0.001 | 4.453 |
| Primary cohort | BMI | Prespecified anthropometric benchmark | 4.009 | 1.720 | 1.322 | 2.117 | <0.001 | — | 6.233 |
| Replication cohort | TyG | No BMI in formula | 0.689 | 0.283 | -0.179 | 0.745 | 0.230 | 0.321 | 0.118 |
| Replication cohort | TG/HDL-C | No BMI in formula | 2.525 | 0.016 | -0.200 | 0.232 | 0.885 | 0.885 | 0.002 |
| Replication cohort | TG/G ratio | No BMI in formula | 0.876 | -0.083 | -0.344 | 0.178 | 0.532 | 0.621 | 0.032 |
| Replication cohort | CHG | No BMI in formula | 0.423 | 0.351 | -0.116 | 0.817 | 0.140 | 0.246 | 0.178 |
| Replication cohort | SPISE | BMI algebraically incorporated | 1.612 | -1.413 | -1.821 | -1.004 | <0.001 | <0.001 | 3.567 |
| Replication cohort | TyG-BMI | BMI algebraically incorporated | 43.964 | 1.421 | 1.006 | 1.835 | <0.001 | <0.001 | 3.510 |
| Replication cohort | METS-IR | BMI algebraically incorporated | 9.012 | 1.473 | 1.032 | 1.914 | <0.001 | <0.001 | 3.349 |
| Replication cohort | BMI | Prespecified anthropometric benchmark | 4.406 | 1.639 | 1.209 | 2.068 | <0.001 | — | 4.294 |

**Note.** Per-IQR scaling is a linear rescaling used to assess effect-size comparability. ΔR² and statistical significance are unchanged by linear rescaling and are not interpreted as an independent test of model fit.

**Table S15. Unindexed LAD sensitivity analyses with additional body-size covariate adjustment**

| **Body-size covariate** | **Cohort** | **Marker** | **Formula class** | **n** | **Adjusted LAD difference per 1-SD (95% CI)** | **Nominal P** | **FDR P** | **ΔR², pp** |
| --- | --- | --- | --- | --- | --- | --- | --- | --- |
| Height | Primary cohort | TyG | No BMI in formula | 788 | 0.339 (0.004, 0.674) | 0.047 | 0.083 | 0.371 |
| Height | Primary cohort | TG/HDL-C | No BMI in formula | 788 | 0.237 (-0.098, 0.573) | 0.166 | 0.232 | 0.181 |
| Height | Primary cohort | TG/G ratio | No BMI in formula | 788 | 0.135 (-0.202, 0.473) | 0.431 | 0.502 | 0.059 |
| Height | Primary cohort | CHG | No BMI in formula | 788 | 0.116 (-0.227, 0.458) | 0.508 | 0.508 | 0.041 |
| Height | Primary cohort | SPISE | BMI algebraically incorporated | 788 | -1.358 (-1.686, -1.030) | <0.001 | <0.001 | 5.727 |
| Height | Primary cohort | TyG-BMI | BMI algebraically incorporated | 788 | 1.247 (0.917, 1.576) | <0.001 | <0.001 | 4.853 |
| Height | Primary cohort | METS-IR | BMI algebraically incorporated | 788 | 1.205 (0.872, 1.538) | <0.001 | <0.001 | 4.467 |
| Height | Primary cohort | BMI | Prespecified anthropometric benchmark | 788 | 1.431 (1.103, 1.759) | <0.001 | — | 6.316 |
| Height | Replication cohort | TyG | No BMI in formula | 813 | 0.191 (-0.175, 0.557) | 0.305 | 0.428 | 0.085 |
| Height | Replication cohort | TG/HDL-C | No BMI in formula | 813 | -0.013 (-0.361, 0.336) | 0.943 | 0.943 | <0.001 |
| Height | Replication cohort | TG/G ratio | No BMI in formula | 813 | -0.133 (-0.484, 0.218) | 0.458 | 0.534 | 0.045 |
| Height | Replication cohort | CHG | No BMI in formula | 813 | 0.229 (-0.146, 0.603) | 0.231 | 0.404 | 0.116 |
| Height | Replication cohort | SPISE | BMI algebraically incorporated | 813 | -1.155 (-1.495, -0.816) | <0.001 | <0.001 | 3.421 |
| Height | Replication cohort | TyG-BMI | BMI algebraically incorporated | 813 | 1.152 (0.811, 1.493) | <0.001 | <0.001 | 3.379 |
| Height | Replication cohort | METS-IR | BMI algebraically incorporated | 813 | 1.123 (0.776, 1.469) | <0.001 | <0.001 | 3.119 |
| Height | Replication cohort | BMI | Prespecified anthropometric benchmark | 813 | 1.273 (0.938, 1.608) | <0.001 | — | 4.221 |
| BSA | Primary cohort | TyG | No BMI in formula | 788 | 0.036 (-0.296, 0.368) | 0.832 | 0.832 | 0.004 |
| BSA | Primary cohort | TG/HDL-C | No BMI in formula | 788 | -0.090 (-0.424, 0.243) | 0.595 | 0.826 | 0.025 |
| BSA | Primary cohort | TG/G ratio | No BMI in formula | 788 | -0.063 (-0.391, 0.266) | 0.708 | 0.826 | 0.012 |
| BSA | Primary cohort | CHG | No BMI in formula | 788 | -0.126 (-0.461, 0.209) | 0.461 | 0.806 | 0.048 |
| BSA | Primary cohort | SPISE | BMI algebraically incorporated | 788 | -0.823 (-1.258, -0.388) | <0.001 | 0.002 | 1.183 |
| BSA | Primary cohort | TyG-BMI | BMI algebraically incorporated | 788 | 0.566 (0.091, 1.041) | 0.020 | 0.068 | 0.475 |
| BSA | Primary cohort | METS-IR | BMI algebraically incorporated | 788 | 0.496 (0.027, 0.964) | 0.038 | 0.089 | 0.375 |
| BSA | Primary cohort | BMI | Prespecified anthropometric benchmark | 788 | 0.948 (0.425, 1.470) | <0.001 | — | 1.090 |
| BSA | Replication cohort | TyG | No BMI in formula | 813 | -0.053 (-0.413, 0.307) | 0.772 | 0.901 | 0.006 |
| BSA | Replication cohort | TG/HDL-C | No BMI in formula | 813 | -0.166 (-0.506, 0.173) | 0.336 | 0.470 | 0.070 |
| BSA | Replication cohort | TG/G ratio | No BMI in formula | 813 | -0.250 (-0.591, 0.091) | 0.150 | 0.470 | 0.157 |
| BSA | Replication cohort | CHG | No BMI in formula | 813 | 0.005 (-0.363, 0.373) | 0.979 | 0.979 | <0.001 |
| BSA | Replication cohort | SPISE | BMI algebraically incorporated | 813 | -0.378 (-0.857, 0.101) | 0.121 | 0.470 | 0.182 |
| BSA | Replication cohort | TyG-BMI | BMI algebraically incorporated | 813 | 0.273 (-0.242, 0.788) | 0.298 | 0.470 | 0.082 |
| BSA | Replication cohort | METS-IR | BMI algebraically incorporated | 813 | 0.254 (-0.252, 0.761) | 0.324 | 0.470 | 0.074 |
| BSA | Replication cohort | BMI | Prespecified anthropometric benchmark | 813 | 0.490 (-0.066, 1.046) | 0.084 | — | 0.226 |

**Note.** All models used unindexed LAD as the outcome and the harmonized core covariates, with height or BSA additionally entered as a covariate. ΔR² denotes the marker-specific incremental R² beyond the corresponding clinical plus body-size covariate model. Benjamini-Hochberg FDR correction was applied to the seven metabolic surrogate indices within each cohort and body-size specification; BMI remained the prespecified benchmark outside that multiplicity family.

**Table S16. AF-phenotype-stratified LAD sensitivity analyses**

| **AF phenotype** | **Cohort** | **Marker** | **Formula class** | **n** | **Adjusted LAD difference per 1-SD (95% CI)** | **Nominal P** | **FDR P** | **ΔR², pp** |
| --- | --- | --- | --- | --- | --- | --- | --- | --- |
| Paroxysmal | Primary cohort | TyG | No BMI in formula | 464 | 0.214 (-0.223, 0.651) | 0.336 | 0.510 | 0.191 |
| Paroxysmal | Primary cohort | TG/HDL-C | No BMI in formula | 464 | 0.058 (-0.366, 0.481) | 0.789 | 0.858 | 0.015 |
| Paroxysmal | Primary cohort | TG/G ratio | No BMI in formula | 464 | -0.037 (-0.439, 0.366) | 0.858 | 0.858 | 0.007 |
| Paroxysmal | Primary cohort | CHG | No BMI in formula | 464 | 0.201 (-0.234, 0.636) | 0.364 | 0.510 | 0.170 |
| Paroxysmal | Primary cohort | SPISE | BMI algebraically incorporated | 464 | -1.125 (-1.555, -0.694) | <0.001 | <0.001 | 5.123 |
| Paroxysmal | Primary cohort | TyG-BMI | BMI algebraically incorporated | 464 | 0.913 (0.488, 1.337) | <0.001 | <0.001 | 3.536 |
| Paroxysmal | Primary cohort | METS-IR | BMI algebraically incorporated | 464 | 0.888 (0.460, 1.315) | <0.001 | <0.001 | 3.302 |
| Paroxysmal | Primary cohort | BMI | Prespecified anthropometric benchmark | 464 | 1.113 (0.684, 1.543) | <0.001 | — | 5.063 |
| Persistent | Primary cohort | TyG | No BMI in formula | 324 | 0.465 (-0.063, 0.994) | 0.084 | 0.116 | 0.922 |
| Persistent | Primary cohort | TG/HDL-C | No BMI in formula | 324 | 0.536 (-0.016, 1.088) | 0.057 | 0.100 | 1.120 |
| Persistent | Primary cohort | TG/G ratio | No BMI in formula | 324 | 0.517 (-0.099, 1.133) | 0.100 | 0.116 | 0.839 |
| Persistent | Primary cohort | CHG | No BMI in formula | 324 | 0.017 (-0.543, 0.577) | 0.952 | 0.952 | 0.001 |
| Persistent | Primary cohort | SPISE | BMI algebraically incorporated | 324 | -1.640 (-2.155, -1.126) | <0.001 | <0.001 | 10.870 |
| Persistent | Primary cohort | TyG-BMI | BMI algebraically incorporated | 324 | 1.724 (1.198, 2.251) | <0.001 | <0.001 | 11.397 |
| Persistent | Primary cohort | METS-IR | BMI algebraically incorporated | 324 | 1.674 (1.140, 2.208) | <0.001 | <0.001 | 10.551 |
| Persistent | Primary cohort | BMI | Prespecified anthropometric benchmark | 324 | 1.845 (1.334, 2.356) | <0.001 | — | 13.507 |
| Paroxysmal | Replication cohort | TyG | No BMI in formula | 409 | 0.409 (-0.117, 0.934) | 0.127 | 0.178 | 0.462 |
| Paroxysmal | Replication cohort | TG/HDL-C | No BMI in formula | 409 | 0.428 (-0.095, 0.951) | 0.108 | 0.178 | 0.513 |
| Paroxysmal | Replication cohort | TG/G ratio | No BMI in formula | 409 | 0.181 (-0.300, 0.662) | 0.459 | 0.536 | 0.109 |
| Paroxysmal | Replication cohort | CHG | No BMI in formula | 409 | 0.122 (-0.377, 0.621) | 0.631 | 0.631 | 0.046 |
| Paroxysmal | Replication cohort | SPISE | BMI algebraically incorporated | 409 | -1.529 (-2.036, -1.021) | <0.001 | <0.001 | 6.425 |
| Paroxysmal | Replication cohort | TyG-BMI | BMI algebraically incorporated | 409 | 1.325 (0.861, 1.789) | <0.001 | <0.001 | 5.822 |
| Paroxysmal | Replication cohort | METS-IR | BMI algebraically incorporated | 409 | 1.346 (0.875, 1.817) | <0.001 | <0.001 | 5.831 |
| Paroxysmal | Replication cohort | BMI | Prespecified anthropometric benchmark | 409 | 1.437 (0.969, 1.905) | <0.001 | — | 6.653 |
| Persistent | Replication cohort | TyG | No BMI in formula | 404 | 0.114 (-0.390, 0.618) | 0.658 | 0.658 | 0.045 |
| Persistent | Replication cohort | TG/HDL-C | No BMI in formula | 404 | -0.124 (-0.588, 0.340) | 0.600 | 0.658 | 0.062 |
| Persistent | Replication cohort | TG/G ratio | No BMI in formula | 404 | -0.231 (-0.742, 0.280) | 0.376 | 0.526 | 0.178 |
| Persistent | Replication cohort | CHG | No BMI in formula | 404 | 0.368 (-0.183, 0.918) | 0.190 | 0.332 | 0.389 |
| Persistent | Replication cohort | SPISE | BMI algebraically incorporated | 404 | -0.906 (-1.364, -0.449) | <0.001 | <0.001 | 3.309 |
| Persistent | Replication cohort | TyG-BMI | BMI algebraically incorporated | 404 | 0.941 (0.444, 1.439) | <0.001 | <0.001 | 3.032 |
| Persistent | Replication cohort | METS-IR | BMI algebraically incorporated | 404 | 0.919 (0.418, 1.420) | <0.001 | <0.001 | 2.857 |
| Persistent | Replication cohort | BMI | Prespecified anthropometric benchmark | 404 | 1.039 (0.560, 1.519) | <0.001 | — | 3.934 |

**Note.** Models were stratified by AF phenotype and therefore omitted AF phenotype from the adjustment set while retaining sex, hypertension, diabetes mellitus, coronary artery disease, prior stroke, and LVEF. Marker Z scores were defined in the full cohort before stratification. ΔR² denotes the marker-specific incremental R² beyond the stratum-specific clinical model. Benjamini-Hochberg FDR correction was applied to the seven metabolic surrogate indices within each cohort-phenotype stratum; BMI remained the prespecified benchmark outside that multiplicity family. AF phenotype is a partial phenotype-based proxy only and does not establish cardiac rhythm at the time of echocardiographic acquisition.

# Supplementary Figures

**Supplementary Figure S1. Metabolic correlation structure across cohorts**

**Supplementary Figure S2. Restricted cubic-spline associations**

**Note.** Curves are displayed over the common central support of each marker across cohorts. Each panel reports P overall and P nonlinearity separately for the primary and replication cohorts; spline knots and exact test output are reported in Table S8.

**Supplementary Figure S3. Exploratory AF-phenotype interactions**

**Note.** No interaction met the prespecified BH-FDR threshold in either cohort; interaction P values and FDR-adjusted P values are reported in Table S9.

**Supplementary Figure S4. Quantile regression across the LAD distribution**

**Note.** A common y-axis is used across markers to preserve comparability of association magnitude.

**Supplementary Figure S5. Secondary diameter-defined LAE discrimination and calibration**

**Note.** LAE is a dichotomized representation of the same LAD measurement and is not treated as an independent endpoint.

**Supplementary Figure S6. Bootstrap ranking stability**

**Note.** Ranking is shown for continuous-LAD association metrics (absolute standardized β and ΔR²); ΔAUC ranking for secondary LAE is reported in Table S12.

**Supplementary Figure S7. Replication-cohort covariate-adjustment sensitivity**

**Note.** The harmonized core model is compared with the same model additionally adjusted for smoking and drinking.
